# Prognostic Implications of Fractional Flow Reserve After Coronary Stenting: A Systematic Review and Meta-analysis

**DOI:** 10.1001/jamanetworkopen.2022.32842

**Published:** 2022-09-22

**Authors:** Doyeon Hwang, Bon-Kwon Koo, Jinlong Zhang, Jiesuck Park, Seokhun Yang, Minsang Kim, Jun Pil Yun, Joo Myung Lee, Chang-Wook Nam, Eun-Seok Shin, Joon-Hyung Doh, Shao-Liang Chen, Tsunekazu Kakuta, Gabor G. Toth, Zsolt Piroth, Nils P. Johnson, Nico H. J. Pijls, Abdul Hakeem, Barry F. Uretsky, Yohei Hokama, Nobuhiro Tanaka, Hong-Seok Lim, Tsuyoshi Ito, Akiko Matsuo, Lorenzo Azzalini, Massoud A. Leesar, Tara Neleman, Nicolas M. van Mieghem, Roberto Diletti, Joost Daemen, Damien Collison, Carlos Collet, Bernard De Bruyne

**Affiliations:** 1 Department of Internal Medicine and Cardiovascular Center, Seoul National University Hospital, Seoul, Korea; 2 Department of Cardiology, The Second Affiliated Hospital, School of Medicine, Zhejiang University, Hangzhou, China; 3 Division of Cardiology, Department of Internal Medicine, Heart Vascular Stroke Institute, Samsung Medical Center, Sungkyunkwan University School of Medicine, Seoul, Korea; 4 Department of Medicine, Keimyung University Dongsan Medical Center, Daegu, Korea; 5 Division of Cardiology, Ulsan Hospital, Ulsan, Korea; 6 Department of Medicine, Inje University Ilsan Paik Hospital, Goyang, Korea; 7 Division of Cardiology, Nanjing First Hospital, Nanjing Medical University, Nanjing, China; 8 Division of Cardiovascular Medicine, Tsuchiura Kyodo General Hospital, Ibaraki, Japan; 9 University Heart Centre Graz, Medical University Graz, Austria; 10 Gottsegen Hungarian Institute of Cardiology, Budapest, Hungary; 11 Weatherhead PET Center For Preventing and Reversing Atherosclerosis, Division of Cardiology, Department of Medicine, University of Texas Medical School and Memorial Hermann Hospital, Houston; 12 Department of Cardiology, Catharina Hospital, Eindhoven, the Netherlands; 13 Division of Cardiovascular Diseases & Hypertension, Robert Wood Johnson Medical School, Rutgers University, New Brunswick, New Jersey; 14 National Institute of Cardiovascular Diseases, Karachi, Pakistan; 15 Central Arkansas VA Health System, Little Rock, Arkansas; 16 University of Arkansas for Medical Sciences, Little Rock; 17 Department of Cardiology, Tokyo Medical University Hachioji Medical Center, Tokyo, Japan; 18 Department of Cardiology, Ajou University School of Medicine, Suwon, Korea; 19 Department of Cardiology, Nagoya City University Graduate School of Medical Sciences, Nagoya, Japan; 20 Department of Cardiology, Kyoto Second Red Cross Hospital, Kyoto, Japan; 21 Division of Cardiology, Department of Medicine, University of Washington, Seattle; 22 Division of Cardiovascular Diseases, University of Alabama, Birmingham; 23 Department of Interventional Cardiology, Thoraxcenter, Erasmus University Medical Centre, Rotterdam, the Netherlands; 24 West of Scotland Regional Heart and Lung Centre, Golden Jubilee National Hospital, Glasgow, United Kingdom; 25 Cardiovascular Center Aalst, Aalst, Belgium; 26 Department of Cardiology, University of Lausanne, Switzerland

## Abstract

**Importance:**

Fractional flow reserve (FFR) after percutaneous coronary intervention (PCI) is generally considered to reflect residual disease. Yet the clinical relevance of post-PCI FFR after drug-eluting stent (DES) implantation remains unclear.

**Objective:**

To evaluate the clinical relevance of post-PCI FFR measurement after DES implantation.

**Data Sources:**

MEDLINE, Embase, and the Cochrane Central Register of Controlled Trials were searched for relevant published articles from inception to June 18, 2022.

**Study Selection:**

Published articles that reported post-PCI FFR after DES implantation and its association with clinical outcomes were included.

**Data Extraction and Synthesis:**

Patient-level data were collected from the corresponding authors of 17 cohorts using a standardized spreadsheet. Meta-estimates for primary and secondary outcomes were analyzed per patient and using mixed-effects Cox proportional hazard regression with registry identifiers included as a random effect. All processes followed the Preferred Reporting Items for Systematic Review and Meta-analysis of Individual Participant Data.

**Main Outcomes and Measures:**

The primary outcome was target vessel failure (TVF) at 2 years, a composite of cardiac death, target vessel myocardial infarction (TVMI), and target vessel revascularization (TVR). The secondary outcome was a composite of cardiac death or TVMI at 2 years.

**Results:**

Of 2268 articles identified, 29 studies met selection criteria. Of these, 28 articles from 17 cohorts provided data, including a total of 5277 patients with 5869 vessels who underwent FFR measurement after DES implantation. Mean (SD) age was 64.4 (10.1) years and 4141 patients (78.5%) were men. Median (IQR) post-PCI FFR was 0.89 (0.84-0.94) and 690 vessels (11.8%) had a post-PCI FFR of 0.80 or below. The cumulative incidence of TVF was 340 patients (7.2%), with cardiac death or TVMI occurring in 111 patients (2.4%) at 2 years. Lower post-PCI FFR significantly increased the risk of TVF (adjusted hazard ratio [HR] per 0.01 FFR decrease, 1.04; 95% CI, 1.02-1.05; *P* < .001). The risk of cardiac death or MI also increased inversely with post-PCI FFR (adjusted HR, 1.03; 95% CI, 1.00-1.07, *P* = .049). These associations were consistent regardless of age, sex, the presence of hypertension or diabetes, and clinical diagnosis.

**Conclusions and Relevance:**

Reduced FFR after DES implantation was common and associated with the risks of TVF and of cardiac death or TVMI. These results indicate the prognostic value of post-PCI physiologic assessment after DES implantation.

## Key Points

**Question:**

What is the clinical relevance of fractional flow reserve (FFR) after percutaneous coronary intervention (PCI) with a drug-eluting stent?

**Findings:**

In this systematic review and individual patient-level meta-analysis, low post-PCI FFR was common and demonstrated a significant and inverse association with target vessel failure. This association remained consistent for the risk of cardiac death or target vessel myocardial infarction.

**Meaning:**

These results support the importance of post-PCI physiologic assessment and the role of post-PCI FFR as a procedural quality metric.

## Teaser

This systematic review and meta-analysis examines clinical outcomes associated with low fractional flow reserve measures after percutaneous coronary interventions with drug-eluting stents.

## Introduction

Fractional flow reserve (FFR) expresses the reduction in maximal flow due to coronary disease and has become the standard used to select treatment strategy.^1,2,3^ Percutaneous coronary intervention (PCI) does not guarantee optimal revascularization, ie, restoration of normal epicardial conductance. Studies have shown that residual pressure gradients remain in 10% to 36% of cases despite angiographically successful PCI.^4,5,6,7^ As FFR measured after PCI is often markedly lower than 1.0, even with an angiographically satisfactory appearance,^4,5,6,7,8^ it has been suggested that low post-PCI FFR should trigger additional interventions.^4,6,8,9,10^ However, not only is the relevance and association of post-PCI FFR with hard outcomes controversial but there is also considerable variation in its reported distribution. Therefore, we performed a systematic review and patient-level meta-analysis to address these clinical uncertainties of post-PCI FFR.

## Methods

### Data Sources and Study Selection

In this systematic review and individual patient-level meta-analysis, MEDLINE, Embase, and the Cochrane Central Register of Controlled Trials were searched for relevant articles regarding post-PCI FFR from inception to June 18, 2022 (eAppendix 1 in the Supplement). This database search was complemented by a manual search of references cited by recent reviews, editorials, and meta-analyses. No restriction was imposed on the study period or sample size, but only English-language articles were considered. Keywords included *post*, *after*, *PCI*, *percutaneous coronary intervention*, *coronary stenting*, *stenting*, *stent*, *stent implantation*, *FFR*, and *fractional flow reserve*. Articles were included if they met the following prespecified criteria: (1) PCI with drug-eluting stents (DES); (2) post-PCI FFR measured at the end of the procedure; (3) clinical follow-up of at least 6 months; (4) clinical outcome data including all-cause death, cardiac death, target vessel myocardial infarction (TVMI), and target vessel revascularization (TVR). Two independent investigators (M.K. and J.P.Y.) screened titles and abstracts, identified duplicates, performed full-article reviews, and determined inclusion. A third investigator (J.P.) supervised the search and adjudicated disagreements. The study protocol was approved by the ethics committee of Seoul National University Hospital and conducted according to the principles of the Declaration of Helsinki. Informed consent requirements were waived as deidentified data were retrospectively collected. All processes followed the Preferred Reporting Items for Systematic Review and Meta-analysis of Individual Participant Data (PRISMA-IPD).^11^ The study protocol was prespecified and registered with PROSPERO (CRD42021234748).

### Data Collection and Merging

The principal investigator of each study was contacted to provide anonymized, patient-level data. If there were multiple articles from the same cohort, we asked for data with the largest number of included patients. Demographics (age and sex), clinical risk factors (hypertension, diabetes, hypercholesterolemia, current smoking, clinical diagnosis, and previous MI), and catheterization data (angiographic and physiologic) were aggregated using standardized definitions for variables. Race and ethnicity were not included in demographics because data were not available. A central monitoring team at Seoul National University Hospital (D.H. and J.P.) double-checked all submitted data.

### Study Population and Outcomes

The pooled population comprised patients who underwent PCI with DES plus post-PCI FFR measured at the end of the procedure. Each study population was followed for at least 6 months for clinical outcomes. In patients with multivessel interrogation, the single vessel with the lowest post-PCI FFR value was chosen.

The primary clinical outcome was target vessel failure (TVF), defined as a composite of cardiac death, TVMI, and TVR over 2 years. The secondary clinical outcome was a composite of cardiac death or TVMI. All deaths were considered cardiac in origin unless a noncardiac cause was indicated. TVMI was defined as a myocardial infarction that occurred by any lesion in the same target vessel. TVR was defined as any repeat revascularization in the target vessel.

### Quality Assessment

Included studies were evaluated for quality using the Newcastle-Ottawa scale.^12^ Each study was assessed based on an 8-point scale using 3 criteria: selection process (3 points), comparability (2 points), and outcome (3 points). Two investigators (D.H. and J.P.) assessed the risk of bias. We did not exclude individual studies from the analysis based on the risk of bias assessment.

### Statistical Analysis

Categorical variables were summarized as counts and percentages, and continuous variables as mean or median averages according to data distribution. All analyses were performed in a per-patient manner with 1-stage meta-analyses. The cumulative incidence of clinical outcomes was estimated using Kaplan-Meier estimates at 2 years, and a log-rank test was used to compare group differences. Hazard ratio (HR) and 95% CIs were calculated from a mixed-effects Cox proportional hazard regression with registry identifiers included as a random effect. Heterogeneity was assessed by the estimated variance of random effects (τ^2^). The proportional hazards assumption was evaluated by Schoenfeld residuals. For calculating the multivariable-adjusted HR and its 95% CI, the following variables were included in the Cox proportional hazards regression model: age, sex, hypertension, diabetes, hypercholesterolemia, and clinical diagnosis. Estimated TVF risk at 2 years derived from the multivariable-adjusted Cox proportional hazard regression model was plotted against post-PCI FFR value, and a locally weighted scatterplot smoothing regression line was used to explore the prognostic value of post-PCI FFR. The optimal cutoff value of post-PCI FFR for outcomes was calculated based on maximizing the difference of log-rank statistics. Subgroups analyzed for sensitivity included: age 65 years and older, sex, hypertension, diabetes, and acute coronary syndrome. All applicable *P* values were 2-sided, and *P* < .05 was considered statistically significant. The software package R version 4.0.3 (R Foundation for Statistical Computing) was used for statistical analysis.

## Results

From MEDLINE, Embase, and the Cochrane Central Register of Controlled Trials, a total of 2268 studies were identified, and 108 studies underwent full-article review (eFigure 1 in the Supplement). Twenty-nine articles^13,14,15,16,17,18,19,20,21,22,23,24,25,26,27,28,29,30,31,32,33,34,35,36,37,38,39,40,41^ met the inclusion criteria, and corresponding authors were contacted for data sharing (eTable 1 in the Supplement). Except for one study, the corresponding authors of 28 studies from 17 cohorts agreed to provide anonymized or deidentified data for this individual patient-level meta-analysis (eTables 2 through 4 in the Supplement). Cohorts were geographically diverse and located across 16 countries. The quality of included studies and cohorts was assessed by the Newcastle-Ottawa scale. Most cohorts showed high quality except for 2 cohorts with moderate quality (eTable 5 in the Supplement).

**Figure 1.**
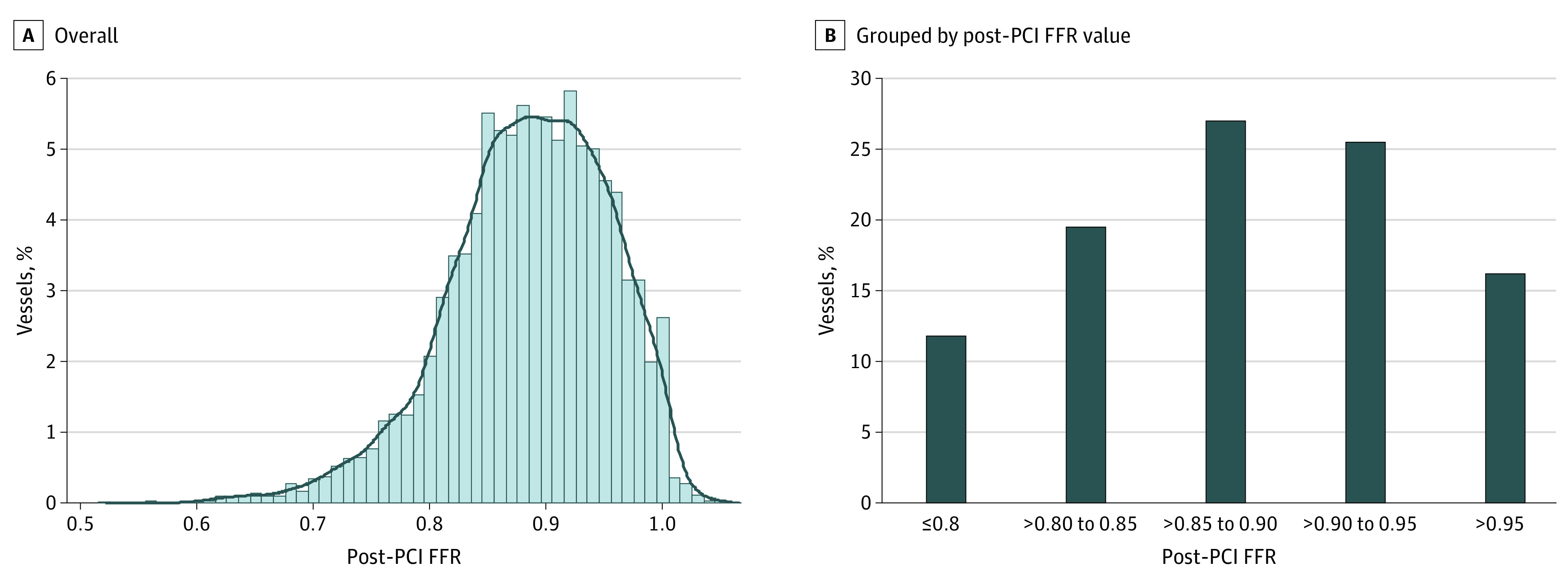
Distribution of Post-PCI FFR FFR indicates fractional flow reserve; PCI, percutaneous coronary intervention.

After checking data integrity and excluding patients with balloon angioplasty or bare-metal stent implantation, a total of 5277 patients with 5869 vessels were included in this study (eFigure 1 in the Supplement). Mean (SD) age was 64.4 (10.1) years and 4141 patients (78.5%) were men. The target vessel was the left anterior descending artery in 3565 patients (67.8%) (eTable 6 in the Supplement). The median (IQR) value of post-PCI FFR was 0.89 (0.84-0.94) in 5869 vessels. Post-PCI FFR greater than 0.95 was achieved in 16.2%, 0.90 or below in 58.3%, and 0.80 or below in 11.8% of 5869 vessels (Figure 1; eTable 7 in the Supplement).

**Figure 2.**
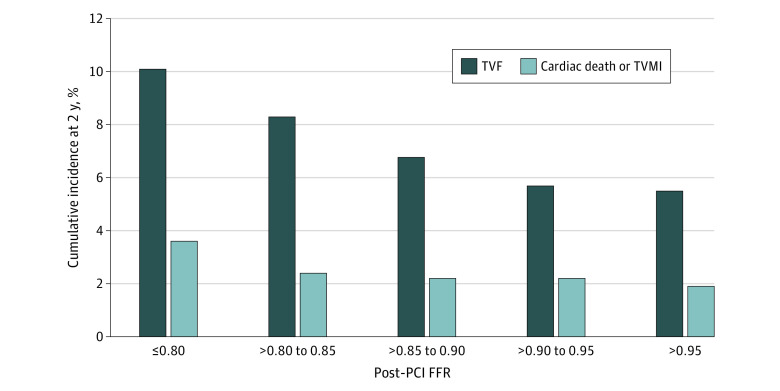
Event Rates According to Post-PCI FFR FFR indicates fractional flow reserve; PCI, percutaneous coronary intervention; TVF, target vessel failure; TVMI, target vessel myocardial infarction.

At 2-year follow-up, TVF occurred in 340 patients (7.2%) and cardiac death or TVMI in 111 patients (2.4%) (Table). The cumulative incidence of TVF at 2 years was 10.1%, 8.3%, 7.1%, 6.1% and 5.5% in patients with post-PCI FFR of 0.80 or below, 0.81 to 0.85, 0.86 to 0.90, 0.91 to 0.95, and above 0.95, respectively. Cardiac death or TVMI occurred with an incidence of 3.6%, 2.4%, 2.2%, 2.2%, and 1.9% in patients with post-PCI FFR of 0.80 or below, 0.81 to 0.85, 0.86 to 0.90, 0.91 to 0.95, and above 0.95, respectively (Figure 2). Taking patients with post-PCI FFR above 0.95 as a reference, the adjusted HR for TVF in patients with post-PCI FFR of 0.80 or below was 2.11 (95% CI, 1.39-3.21; *P* < .001), and that for cardiac death or TVMI was 2.56 (95% CI, 1.12-5.87; *P* = .03) (eFigure 2 and eTable 8 in the Supplement).

**Table.** Risk of Clinical Events at 2 Years per Post-PCI FFR 0.01 Decrease

Event	Total events, No. (%)^a^	HR (95% CI)	*P* value	Adjusted HR (95% CI)^b^	*P* value	τ^2^
Target vessel failure	340/5204 (7.2)	1.034 (1.019-1.049)	<.001	1.035 (1.020-1.051)	<.001	<0.001
Cardiac death or TVMI	111/5204 (2.4)	1.035 (1.002-1.068)	.04	1.034 (1.001-1.068)	.049	<0.001
Cardiac death	64/5274 (1.4)	1.047 (1.013-1.083)	.006	1.045 (1.011-1.081)	.009	0.001
TVMI	57/5207 (1.2)	1.018 (0.973-1.066)	.44	1.018 (0.973-1.066)	.44	0.001
TVR	285/5276 (6.0)	1.033 (1.016-1.051)	<.001	1.034 (1.015-1.052)	<.001	<0.001

Abbreviations: FFR, fractional flow reserve; HR, hazard ratio; PCI, percutaneous coronary intervention; TVMI, target vessel myocardial infarction; TVR, target vessel revascularization.

^a^ The cumulative incidence of clinical outcomes at 2 years is presented as Kaplan-Meier estimates.

^b^ The following patient risk factors were included in the multivariable-adjusted mixed-effects Cox proportional hazard regression model: age, sex, hypertension, diabetes, hypercholesterolemia, and acute coronary syndrome.

**Figure 3.**
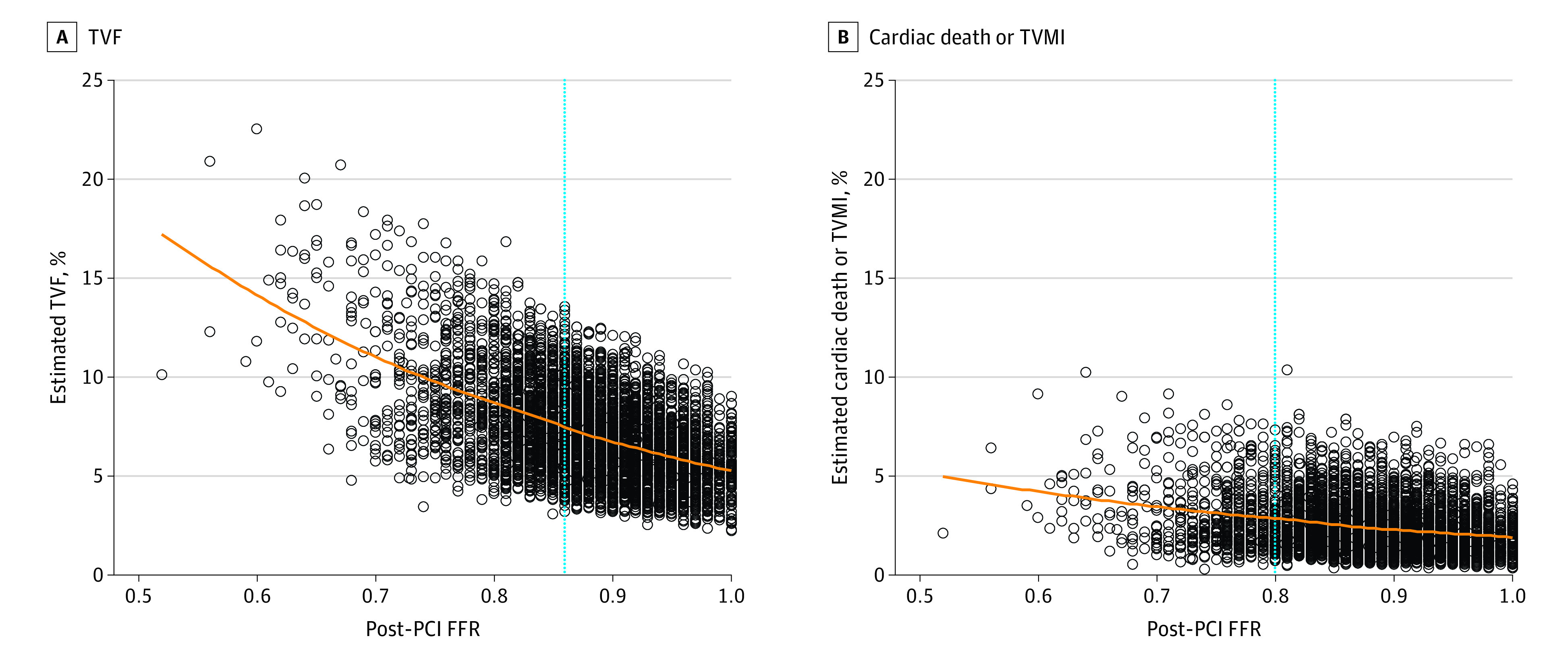
Association Between Post-PCI FFR and Clinical Events The estimated risk of clinical events was calculated from multivariable-adjusted, mixed-effects Cox proportional hazards regression, accounting for age, sex, hypertension, diabetes mellitus, hypercholesterolemia, and clinical diagnosis. Blue dotted lines represent optimal cutoff values for TVF (0.86) and cardiac death or TVMI (0.80) at 2 years. FFR indicates fractional flow reserve; PCI, percutaneous coronary intervention; TVF, target vessel failure; TVMI, target vessel myocardial infarction.

Clinical outcomes had a significant inverse association with post-PCI FFR (Figure 3 and Table). Per 0.01 decrease in post-PCI FFR, the risk of adverse outcomes increased (adjusted HR of TVF, 1.04; 95% CI, 1.02-1.05; *P* < .001; adjusted HR of cardiac death or TVMI, 1.034; 95% CI, 1.00-1.07; *P* = .049). Subgroup analyses showed consistent trends without significant interactions (eFigure 3 and eTable 9 in the Supplement). Post-PCI FFR was independently associated with both TVF and cardiac death or TVMI (eTable 10 in the Supplement).

**Figure 4.**
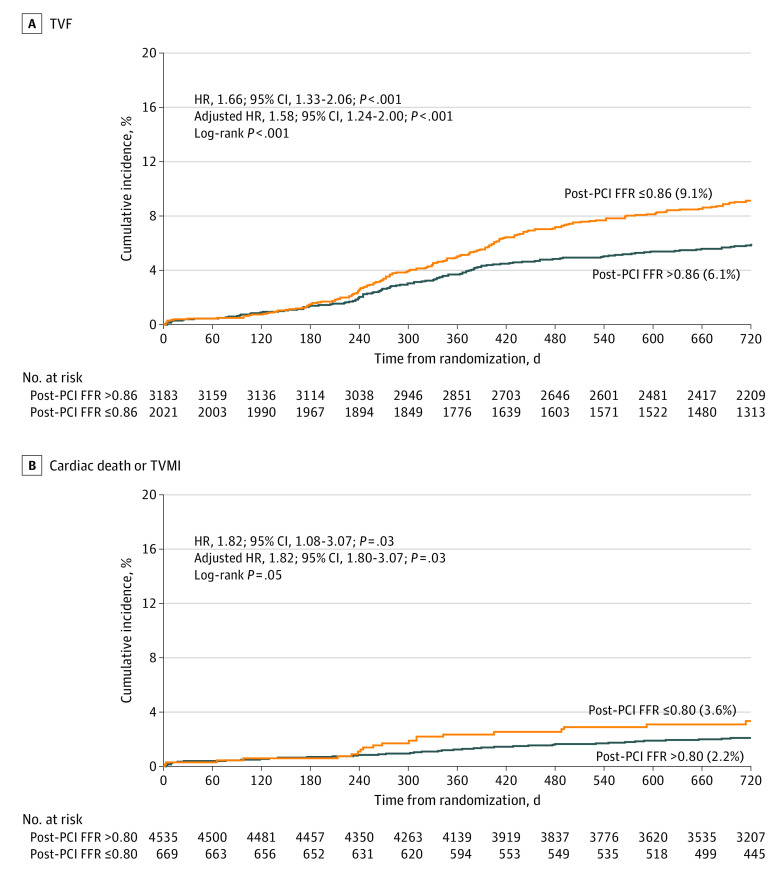
Clinical Events According to Post-PCI FFR Cutoff FFR indicates fractional flow reserve; HR, hazard ratio; PCI, percutaneous coronary intervention; TVF, target vessel failure; TVMI, target vessel myocardial infarction.

Optimal cutoff values of post-PCI FFR were 0.86 for TVF and 0.80 for cardiac death or TVMI (eFigure 4 in the Supplement). The cumulative incidence of TVF at 2 years was significantly higher in patients with post-PCI FFR of 0.86 or below than with post-PCI FFR above 0.86 (9.1% vs 6.1%; adjusted HR, 1.58; 95% CI, 1.24-2.00; *P* < .001) (Figure 4; eTable 11 in the Supplement). The risk of cardiac death or TVMI was also significantly higher in patients with post-PCI FFR of 0.80 or below (3.6% vs 2.2%; adjusted HR, 1.82; 95% CI, 1.08-3.07; *P* = .03) (Figure 4; eTable 11 in the Supplement).

## Discussion

This systematic review and patient-level meta-analysis of post-PCI FFR after DES implantation evaluated a total of 5277 patients and 5869 vessels from 17 cohorts in 28 previous articles across 16 countries. Its results demonstrated that markedly abnormal post-PCI FFR (ie, 0.80 or below) was reported in 11.8% of vessels after DES implantation. A significant, inverse association was observed between post-PCI FFR and the risk of adverse clinical events, consistent among subgroups. Optimal cutoff values for post-PCI FFR were 0.86 for TVF and 0.80 for cardiac death or TVMI, significantly differentiating risk over 2 years.

The presence of myocardial ischemia is one of the important prognostic factors in patients with CAD.^42^ Coronary revascularization is performed to relieve ischemia, thereby reducing future cardiovascular events.^1,2,3^ The success of PCI is generally assessed by coronary angiography, despite its well-accepted limitations in defining stenosis severity before PCI.^43,44,45,46^ However, previous studies demonstrated that there could be residual ischemia even after angiographically successful PCI.^47,48^ Based on intracoronary physiology assessment, recent studies reported that suboptimal epicardial physiology (often referred to as “residual ischemia”) was found in 10% to 36% of patients.^4,5,6,7,8^ In the current meta-analysis, we found a median (IQR) post-PCI FFR value of 0.89 (0.84-0.94) with more than half of post-PCI FFR of 0.90 or below and 11.8% with post-PCI FFR of 0.80 or below. These results support the importance of post-PCI physiologic assessment, even after angiographically successful PCI.

Post-PCI FFR reflects the degree of maximum flow reduction due to residual disease burden in the coronary artery after revascularization.^9,10^ This flow reduction can originate from suboptimal stent deployment as well as residual disease in the nonstented segment.^9,10^ Various studies have demonstrated the role of post-PCI FFR estimating outcomes after balloon angioplasty, bare-metal stent, or DES implantation, including meta-analyses.^9,10,49,50,51,52,53^ Bech et al^53^ first reported that a post-PCI FFR value of 0.90 was associated with clinical outcomes after balloon angioplasty, and this value was the same after bare-metal stent implantation reported by Pijls et al.^49^ Rimac et al^51^ performed a study-level meta-regression and reported an inverse association between post-PCI FFR and the risk of clinical events, including repeat revascularization and major adverse cardiac events (MACE). Johnson et al^50^ performed a patient-level meta-analysis of 966 patients and also demonstrated the continuous, inverse association between post-PCI FFR and the risk of MACE (HR, 0.86; 95% CI, 0.80-0.93; *P* < .001). However, these previous meta-analyses included many patients with balloon angioplasty or bare-metal stent implantation and thus have less relevance to modern clinical practice. Considering the superior efficacy and safety of DES compared with balloon angioplasty or bare-metal stent, the prognostic value of the same post-PCI FFR values may be different.^54,55,56,57^ The value of post-PCI FFR for risk projection has been revalidated in several studies in the DES era, but there has been scarce evidence for the association between post-PCI FFR and hard outcomes.^9,10^ Two previous studies reported that patients with a lower post-PCI FFR value showed significantly higher rates of cardiac death or TVMI; however, this finding was not reproduced in other studies.^4,58^ In these regards, extensive and dedicated evidence for post-PCI FFR in the DES era is needed, and our current study collected patient-level data to evaluate the value of post-PCI FFR for estimating risk after DES implantation. Although one published study could not be included,^59^ the current results incorporating essentially all available patient-level data worldwide demonstrated a significant and inverse association between post-PCI FFR and the risk of TVF after DES implantation. This association remained consistent for the risk of cardiac death or TVMI. Despite the insignificant interaction *P* values in subgroup analyses, it is interesting to note that the association between post-PCI FFR and clinical events diminished in patients with diabetes. This finding suggests that the prognostic relevance of post-PCI FFR can be influenced by clinical risk factors that affect disease progression, plaque vulnerability, or microvascular dysfunction.

Previous studies have reported different optimal cutoff values of post-PCI FFR. This variation may relate to the effect of factors such as study population, underlying comorbidities, lesion, and procedural characteristics, follow-up duration, and clinical outcomes.^4,51,58,59,60,61,62,63,64,65^ In our study, optimal cutoff values of post-PCI FFR were 0.86 for TVF and 0.80 for cardiac death or TVMI at 2 years. This result supports post-PCI FFR as a procedural quality metric. However, it is still unclear whether low poststent FFR is a correctable risk factor or just a risk marker. Jeremias et al^6^ reported that 80% of lesions with physiologically suboptimal PCI results were untreated focal stenoses amenable to PCI, albeit when using a very liberal definition for focal disease. Bommel et al^5^ reported that more than half of significant pressure drops were found in nonstented segments, although rarely focal. These studies suggest that there may be an opportunity to optimize PCI results using post-PCI physiologic assessment and thereby outcomes. For example, Agarwal et al^4^ demonstrated that additional intervention in patients with suboptimal post-PCI FFR improved the mean (SD) post-PCI FFR value from 0.78 (0.07) to 0.87 (0.05). In addition to risk stratification after PCI, post-PCI FFR reflects residual angiographically inappreciable disease in both stented and nonstented segments. Post-PCI FFR measurement and comprehensive FFR pullback after PCI might reveal these hidden problems and maximize the benefit of PCI. Accordingly, Collison et al^8^ conducted a randomized controlled trial of a post-PCI FFR pullback-guided optimization strategy. Forty of 131 patients (30.5%) of patients in the intervention group were identified as having targets for additional intervention, which increased the mean (SD) post-PCI FFR value in this cohort from 0.76 (0.08) to 0.82 (0.06). There was no significant between-group difference in the study’s primary end point of the proportion of patients with final post-PCI FFR of 0.90 or over 0.90 (intervention minus control, 10%; 95% CI, −1.84 to 21.91; *P* = .10). The proportion of patients with a final FFR of 0.80 or below 0.80 was significantly reduced when compared with the angiography-guided control group (−11.2%; 95% CI, −21.87 to −0.35; *P* = .045).^8^ Further research is still needed to determine whether additional procedures based on post-PCI FFR values can improve patient outcomes, and ongoing clinical studies (FFR REACT^66^ or DEFINE GPS) will provide further information. Intracoronary imaging can be helpful in understanding the reason for low post-PCI FFR value and determining the treatment strategy.^67,68^ In addition, the novel indices derived from the pre-PCI hyperemic pullback tracing may allow for the estimation of the physiologic result after revascularization since focal disease responds to stent implantation better than diffuse disease.^69,70,71^ Application of nonhyperemic pressure pullback and its coregistration with coronary angiography can also help physicians perform physiologically appropriate PCI.^72,73^

### Limitations

This study had several limitations. The results of our analysis should be understood in the context of limitations from pooled, patient-level data. Included studies were performed over a 10-year period, during which time PCI techniques have evolved. The majority of post-PCI FFR values were not masked to the physicians, and this could have been a potential source of bias as TVR was the main contributor to clinical events. Our meta-analysis did not include information on medical therapy or on intravascular imaging use during PCI. There is a possibility results were affected by selection bias because some of the included studies did not mandate post-PCI FFR measurement. FFR measurement was not standardized across the studies. Finally, information on pressure pullback tracing was available in only a small proportion of cases. Therefore, the location and influence of residual pressure gradients on outcomes could not be assessed in this study.

## Conclusions

Low post-PCI FFR values were common after DES implantation, and were independently associated with future risk of TVF and of cardiac death or TVMI. These results indicate prognostic value of post-PCI physiologic assessment in patients after DES implantation.

## Notes

Supplement. **eAppendix 1.** Search Strategy on PubMed, EMBASE, and Cochrane Library **eAppendix 2.** List of Excluded Studies **eTable 1.** List of Studies Met the Criteria for the Post-PCI FLOW Registry **eTable 2.** List of Studies and Cohorts Included in the Post-PCI FLOW Registry **eTable 3.** Number of Patients Provided by Each Cohort and Number of Patients Included in the Master Data Set **eTable 4.** Description of the Included Cohorts **eTable 5.** The Newcastle-Ottawa Scale for Assessing the Quality of Observational Cohorts **eTable 6.** Baseline Characteristics of the Study Population **eTable 7.** Per-vessel Specific Characteristics and Outcomes **eTable 8.** Cumulative Incidence and Risk of Clinical Events According to Post-PCI FFR Strata **eTable 9.** The Risk of Clinical Events at 2 Years per Post-PCI FFR 0.01 Decrease in Subgroups **eTable 10.** Predictors of TVF and Cardiac Death or TVMI **eTable 11.** Clinical Events According to Post-PCI FFR Cut-off Value **eFigure 1.** Flow Chart of Study Selection Process **eFigure 2.** Hazard Ratios of Clinical Events According to Post-PCI FFR Strata **eFigure 3.** The Risks of Clinical Events per Post-PCI FFR 0.01 Decrease According to Subgroups **eFigure 4.** Optimal Cut-off Values of Post-PCI FFR for Predicting Future Events
eReferences.
