# Supplementary material for: Prognostic Implications of Fractional Flow Reserve After Coronary Stenting: A Systematic Review and Meta-analysis
Source: JAMA Netw Open. 2022 Sep 22;5(9):e2232842. doi: 10.1001/jamanetworkopen.2022.32842 (PMC9500557; doi:10.1001/jamanetworkopen.2022.32842)
Supplement: Supplement. — eAppendix 1. Search Strategy on PubMed, EMBASE, and Cochrane Library eAppendix 2. List of Excluded Studies eTable 1. List of Studies Met the Criteria for the Post-PCI FLOW Registry eTable 2. List of Studies and Cohorts Included in the Post-PCI FLOW Registry eTable 3. Number of Patients Provided by Each Cohort and Number of Patients Included in the Master Data Set eTable 4. Description of the Included Cohorts eTable 5. The Newcastle-Ottawa Scale for Assessing the Quality of Observational Cohorts eTable 6. Baseline Characteristics of the Study Population eTable 7. Per-vessel Specific Characteristics and Outcomes eTable 8. Cumulative Incidence and Risk of Clinical Events According to Post-PCI FFR Strata eTable 9. The Risk of Clinical Events at 2 Years per Post-PCI FFR 0.01 Decrease in Subgroups eTable 10. Predictors of TVF and Cardiac Death or TVMI eTable 11. Clinical Events According to Post-PCI FFR Cut-off Value eFigure 1. Flow Chart of Study Selection Process eFigure 2. Hazard Ratios of Clinical Events According to Post-PCI FFR Strata eFigure 3. The Risks of Clinical Events per Post-PCI FFR 0.01 Decrease According to Subgroups eFigure 4. Optimal Cut-off Values of Post-PCI FFR for Predicting Future Events eReferences. [file jamanetwopen-e2232842-s001.pdf]

Supplemental Online Content

Hwang D, Koo BK, Zhang J, et al. Prognostic implications of fractional flow reserve after coronary stenting: a systematic review and meta-analysis. *JAMA Netw Open.* 2022;5(9):e2232842. doi:10.1001/jamanetworkopen.2022.32842

eAppendix 1. Search Strategy on PubMed, EMBASE, and Cochrane Library

eAppendix 2. List of Excluded Studies

eTable 1. List of Studies Met the Criteria for the Post-PCI FLOW Registry

eTable 2. List of Studies and Cohorts Included in the Post-PCI FLOW Registry

eTable 3. Number of Patients Provided by Each Cohort and Number of Patients Included in the Master Data Set

eTable 4. Description of the Included Cohorts

eTable 5. The Newcastle-Ottawa Scale for Assessing the Quality of Observational Cohorts

eTable 6. Baseline Characteristics of the Study Population

eTable 7. Per-vessel Specific Characteristics and Outcomes

eTable 8. Cumulative Incidence and Risk of Clinical Events According to Post-PCI FFR Strata

eTable 9. The Risk of Clinical Events at 2 Years per Post-PCI FFR 0.01 Decrease in Subgroups

eTable 10. Predictors of TVF and Cardiac Death or TVMI

eTable 11. Clinical Events According to Post-PCI FFR Cut-off Value

eFigure 1. Flow Chart of Study Selection Process

eFigure 2. Hazard Ratios of Clinical Events According to Post-PCI FFR Strata

eFigure 3. The Risks of Clinical Events per Post-PCI FFR 0.01 Decrease According to Subgroups

eFigure 4. Optimal Cut-off Values of Post-PCI FFR for Predicting Future Events

eReferences.

© 2022 Hwang D et al. *JAMA Network Open.*

This supplemental material has been provided by the authors to give readers additional information about their work.

© 2022 Hwang D et al. *JAMA Network Open.*

eAppendix 1. Search Strategy on PubMed, EMBASE, and Cochrane Library

**Pubmed**			**EMBASE**			**Cochrane Library**		
**#14**	#3 and #10 and #13	**599**	**#14**	#3 and #10 and #13	**1,438**	**#14**	#3 and #10 and #13	**231**
**#13**	#11 or #12	**4,974**	**#13**	#11 or #12	**10,474**	**#13**	#11 or #12	**709**
**#12**	Fractional flow reserve	**3,687**	**#12**	Fractional flow reserve	**8,631**	**#12**	Fractional flow reserve	**561**
**#11**	FFR	**3,725**	**#11**	FFR	**6,722**	**#11**	FFR	**525**
**#10**	#4 or #5 or #6 or #7 or #8 or #9	**138,442**	**#10**	#4 or #5 or #6 or #7 or #8 or #9	**233,585**	**#10**	#4 or #5 or #6 or #7 or #8 or #9	**25,326**
**#9**	Stent implantation	**10,645**	**#9**	Stent implantation	**17,841**	**#9**	Stent implantation	**3,532**
**#8**	stent	**83,586**	**#8**	stent	**137,084**	**#8**	stent	**14,125**
**#7**	stenting	**35,275**	**#7**	stenting	**59,534**	**#7**	stenting	**5,584**
**#6**	Coronary stenting	**2,999**	**#6**	Coronary stenting	**4,312**	**#6**	Coronary stenting	**2,894**
**#5**	Percutaneous coronary intervention	**38,982**	**#5**	Percutaneous coronary intervention	**61,961**	**#5**	Percutaneous coronary intervention	**11,803**
**#4**	PCI	**31,474**	**#4**	PCI	**66,948**	**#4**	PCI	**9,523**
**#3**	#1 or #2	**6,031,609**	**#3**	#1 or #2	**8,312,694**	**#3**	#1 or #2	**837,188**
**#2**	after	**5,447,234**	**#2**	after	**7,434,051**	**#2**	after	**739,447**
**#1**	post	**979,209**	**#1**	post	**1,591,751**	**#1**	post	**213,121**

© 2022 Hwang D et al. *JAMA Network Open.*

eAppendix 2. List of Excluded Studies

i. Studies with inadequate end points

1. Fujita H, Inoue N, Matsuo Y, et al. Fractional myocardial flow reserve (FFRmyo) after coronary intervention as a predictor of chronic restenosis. J Invasive Cardiol 1999; 11(9): 527-32.

2. Nakamura S, Anzai H, Takagi T, et al. [Pressure wire guide provisional coronary stent implantation]. J Cardiol 2001; 37(4): 191-9.

3. Pijls NH, Klauss V, Siebert U, et al. Coronary pressure measurement after stenting predicts adverse events at follow-up: a multicenter registry. Circulation 2002; 105(25): 2950-4.

4. Tanaka N, Takazawa K, Shindo N, et al. Decrease of fractional flow reserve shortly after percutaneous coronary intervention. Circ J 2006; 70(10): 1327-31.

5. Ntalianis A, Sels JW, Davidavicius G, et al. Fractional flow reserve for the assessment of nonculprit coronary artery stenoses in patients with acute myocardial infarction. JACC Cardiovasc Interv 2010; 3(12): 1274-81.

6. Rai A, Bahremand M, Saidi MR, et al. The Value of Pre- and post-stenting fractional flow reserve for predicting mid-term stent restenosis following percutaneous coronary intervention (PCI). Glob J Health Sci 2015; 8(7): 240-44.

7. Baranauskas A, Peace A, Kibarskis A, et al. FFR result post PCI is suboptimal in long diffuse coronary artery disease. EuroIntervention 2016; 12(12): 1473-80.

8. Murai T, Yonetsu T, Kanaji Y, et al. Prognostic value of the index of microcirculatory resistance after percutaneous coronary intervention in patients with non-ST-segment elevation acute coronary syndrome. Catheter Cardiovasc Interv 2018; 92(6): 1063-74.

9. Nakamura D, Wijns W, Price MJ, et al. New Volumetric analysis method for stent expansion and its correlation with final fractional flow reserve and clinical outcome: An ILUMIEN I Substudy. JACC Cardiovasc Interv 2018; 11(15): 1467-78.

10. Zhao Q, Ji Z, Li X, et al. Analysis of the clinical value of fractional flow reserve for prognosis evaluation of patients of percutaneous coronary intervention. Exp Ther Med 2018; 15(1): 673-8.

ii. Study with a follow-up less than 6 months

1. van Bommel RJ, Masdjedi K, Diletti R, et al. Routine fractional flow reserve measurement after percutaneous coronary intervention. Circ Cardiovasc Interv 2019; 12(5): e007428.

iii. Inappropriate study design or study population

1. Bech GJ, De Bruyne B, Akasaka T, et al. Coronary pressure and FFR predict long-term outcome after PTCA. Int J Cardiovasc Intervent 2001; 4(2): 67-76.

2. van't Veer M, Pijls NH, Aarnoudse W, Koolen JJ, van de Vosse FN. Evaluation of the haemodynamic characteristics of drug-eluting stents at implantation and at follow-up. Eur Heart J 2006; 27(15): 1811-7.

3. Beleslin B, Ostojic M, Djordjevic-Dikic A, et al. The value of fractional and coronary flow reserve in predicting myocardial recovery in patients with previous myocardial infarction. Eur Heart J 2008; 29(21): 2617-24.

4. Samady H, McDaniel M, Veledar E, et al. Baseline fractional flow reserve and stent diameter predict optimal post-stent fractional flow reserve and major adverse cardiac events after bare-metal stent deployment. JACC Cardiovasc Interv 2009; 2(4): 357-63.

© 2022 Hwang D et al. *JAMA Network Open.*

5. Ye F, Zhang JJ, Tian NL, et al. The acute changes of fractional flow reserve in DK (double kissing), crush, and 1-stent technique for true bifurcation lesions. J Interv Cardiol 2010; 23(4): 341-5.

6. Brito MB, Sant'Anna FM, Soares Jr RSP, Couceiro SLM, Buczynski LC, Barrozo CAM. Use of myocardial fractional flow reserve to identify predictors of poor prognosis after percutaneous coronary interventions. Revista Brasileira de Cardiologia Invasiva 2013; 21(4): 4.

7. Morris PD, Ryan D, Morton AC, et al. Virtual fractional flow reserve from coronary angiography: modeling the significance of coronary lesions: results from the VIRTU-1 (VIRTUal Fractional Flow Reserve From Coronary Angiography) study. JACC Cardiovasc Interv 2013; 6(2): 149-57.

8. Murai T, Lee T, Yonetsu T, Isobe M, Kakuta T. Influence of microvascular resistance on fractional flow reserve after successful percutaneous coronary intervention. Catheter Cardiovasc Interv 2015; 85(4): 585-92.

9. Kimura Y, Tanaka N, Okura H, et al. Characterization of real-world patients with low fractional flow reserve immediately after drug-eluting stents implantation. Cardiovasc Interv Ther 2016; 31(1): 29-37.

10. Kobayashi Y, Nam CW, Tonino PA, et al. The Prognostic value of residual coronary stenoses after functionally complete revascularization. J Am Coll Cardiol 2016; 67(14): 1701-11.

11. Sakoda K, Tanaka N, Hokama Y, et al. Association of moderate chronic kidney disease with insufficient improvement of fractional flow reserve after stent implantation. Catheter Cardiovasc Interv 2016; 88(2): E38-44.

12. Agarwal SK, Kasula S, Almomani A, et al. Clinical and angiographic predictors of persistently ischemic fractional flow reserve after percutaneous revascularization. Am Heart J 2017; 184: 10-6.

13. Ahn JM, Park DW, Shin ES, et al. Fractional flow reserve and cardiac events in coronary artery disease: data from a prospective IRIS-FFR Registry (Interventional Cardiology Research Incooperation Society Fractional Flow Reserve). Circulation 2017; 135(23): 2241-51.

14. Kawase Y, Omori H, Kawasaki M, et al. Postocclusional hyperemia for fractional flow reserve after percutaneous coronary intervention. Circ Cardiovasc Interv 2017; 10(12):e005674. doi: 10.1161/CIRCINTERVENTIONS.117.005674.

15. Murai T, Kanaji Y, Yonetsu T, et al. Preprocedural fractional flow reserve and microvascular resistance predict increased hyperaemic coronary flow after elective percutaneous coronary intervention. Catheter Cardiovasc Interv 2017; 89(2): 233-42.

16. Pyxaras SA, Toth GG, Di Gioia G, et al. Anatomical and functional assessment of Tryton bifurcation stent before and after final kissing balloon dilatation: Evaluations by three-dimensional coronary angiography, optical coherence tomography imaging and fractional flow reserve. Catheter Cardiovasc Interv 2017; 90(1): E1-e10.

17. Yu Y, Zhou Y, Ma Q, et al. The conical stent in coronary artery improves hemodynamics compared with the traditional cylindrical stent. Int J Cardiol 2017; 227: 166-71.

18. Kawase Y, Kawasaki M, Kikuchi J, et al. Residual pressure gradient across the implanted stent: An important factor of post-PCI physiological results. J Cardiol 2018; 71(5): 458-63.

19. Mohdnazri SR, Karamasis GV, Al-Janabi F, et al. The impact of coronary chronic total occlusion percutaneous coronary intervention upon donor vessel fractional flow reserve and instantaneous wave-free ratio: Implications for physiology-guided PCI in patients with CTO. Catheter Cardiovasc Interv 2018; 92(3): E139-e48.

20. Wolfrum M, De Maria GL, Benenati S, et al. What are the causes of a suboptimal FFR after coronary stent deployment? Insights from a consecutive series using OCT imaging. EuroIntervention 2018; 14(12): e1324-e31.

21. Lee CH, Choi SW, Hwang J, et al. 5-year outcomes according to FFR of left circumflex coronary artery after left main crossover stenting. JACC Cardiovasc Interv 2019; 12(9): 847-55.

© 2022 Hwang D et al. *JAMA Network Open.*

22. van der Hoeven NW, Janssens GN, de Waard GA, et al. Temporal changes in coronary hyperemic and resting hemodynamic indices in nonculprit vessels of patients with ST-segment elevation myocardial infarction. JAMA Cardiol 2019; 4(8): 736-744.

23. Zhang YH, Li J, Flammer AJ, et al. Long-term outcomes after fractional flow reserve-guided percutaneous coronary intervention in patients with severe coronary stenosis. J Geriatr Cardiol 2019; 16(4): 329-37.

24. Ahn SG, Hong S, Son JW, et al. Validation of post-stenting fractional flow reserve with intravascular ultrasound parameters for optimal stent deployment. Int J Cardiovasc Imaging 2020; 36(2): 197-203.

25. Belguidoum S, Meneveau N, Motreff P, et al. Relationship between stent expansion and post-PCI fractional flow reserve: a DOCTORS sub study. EuroIntervention 2020; EIJ-D-19-01103. doi: 10.4244/EIJ-D-19-01103.

iv. Not original articles

1. Chamuleau SA. Fractional flow reserve: can it predict adverse events accurately after coronary stenting? Nat Clin Pract Cardiovasc Med 2005; 2(6): 282-3.

2. Klauss V, Erdin P, Rieber J, et al. Fractional flow reserve for the prediction of cardiac events after coronary stent implantation: results of a multivariate analysis. Heart 2005; 91(2): 203-6.

3. Michels M, Werner H, Onderwater E, van der Giessen WJ. Residual angina pectoris after percutaneous coronary intervention for acute coronary syndrome. Neth Heart J 2005; 13(9): 315-7.

4. Ishibashi Y, Yamauchi M, Uematsu M, et al. The value of fractional flow reserve in predicting myocardial protective effect in patients with previous myocardial infarction undergoing percutaneous coronary intervention. Eur Heart J 2011; 32: 858.

5. Lim HS, Tahk SJ, Yang HM, et al. Usefulness of a trans-stent fractional flow reserve gradient for assessing the immediate results of drug-eluting stent implantation. Eur Heart J 2011; 32: 858.

6. Lim HS, Tahk SJ, Yang HM, et al. Usefulness of a trans-stent fractional flow reserve gradient for assessing the results of drug-eluting stent implantation. Catheter Cardiovasc Interv 2011; 77: S69-S70.

7. Lim HS, Tahk SJ, Yang HM, et al. Comparison of fractional flow reserve and intravascular ultrasound for predicting long-term clinical outcomes after drug-eluting stent implantation. Eur Heart J 2012; 33: 9.

8. Ahn SG, Lee JH, Lee JW, Youn YJ, Lee SH, Yoon J. Relationship between IVUS parameters and post-DES FFR. EuroIntervention 2013; 9: 122.

9. Ando H, Takashima H, Fujimoto M, et al. Impact of post-stent fractional flow reserve on long-term adverse event after drug-eluting stent implantation. J Am Coll Cardiol 2013; 62(18): B185.

10. Kim HY, Doh JH, Choi WH, Nam CW, Koo BK, Lee SY. Influence of post-stent fractional flow reserve and stent cross-sectional area on clinical outcome in patient treated with drug eluting stent. Circulation 2013; 128(22).

11. Ando H, Takashima H, Suzuki A, et al. Fractional flow reserve for the prediction of cardiac events after drug-eluting stent implantation. Eur Heart J 2014; 35: 1139.

12. Ando H, Takashima H, Suzuki A, et al. Lesion characteristics as a predictor of optimal post-stent fractional flow reserve. Eur Heart J 2014; 35: 1141.

13. Ando H, Takashima H, Suzuki A, et al. Impact of pressure drop pattern on post-stent fractional flow reserve. Catheter Cardiovasc Interv 2014; 83: S96.

14. Bravo Baptista S, Raposo L, Santos L, et al. Patterns of use and clinical utility of FFR in patients referred for coronary angiography: The POST-IT (POrtuguese Study on the Evaluation of FFR-guIded Treatment of coronary disease) prospective multicentre registry. EuroIntervention 2014.

© 2022 Hwang D et al. *JAMA Network Open.*

15. Banerjee S. Provisional observation of FFR outcome proves utility in ambiguous vessel abnormality. Indian Heart Journal 2015; 67: S67-S8.

16. Baranauskas A, Davidavicius G, Bajoras V, Kibarskis A, Bilkis V, Laucevicius A. Predictive FFR value after PCI on long coronary lesions. JACC Cardiovasc Interv 2015; 8(2): S23.

17. Hakeem A, Agarwal SK, Kasula S, Hacioglu Y, Ahmed Z, Uretsky B. Value of routine post PCI FFR in the identification and management of angiographically optimized stented lesions. J Am Coll Cardiol 2015; 65(10): A1860.

18. Kasula S, Agarwal SK, Hacioglu Y, Uretsky B, Hakeem A. Clinical and prognostic value of post stenting FFR for assessment of ischemia reduction in patients with acute coronary syndromes. J Am Coll Cardiol 2015; 65(10): A1805.

19. Kikuchi J, Matsuo H, Kawase Y, et al. Evaluation of physiological improvement after PCI using instantaneous wave-free ratio (iFR): The potential of iFR to detect and predict post-PCI residual ischaemia. EuroIntervention 2015.

20. Baranauskas A, Peace A, Kibarskis A, et al. FFR result post-PCI is suboptimal in long diffuse coronary artery disease treated with newer-generation DES. EuroIntervention 2016: 107.

21. Matsuda J, Ichijo S, Hamaya R, et al. The discordance between FFR and CFR changes after percutaneous coronary intervention. Eur Heart J 2016; 37: 872.

22. Murai T, Yonetsu T, Kanaji Y, et al. Preprocedural fractional flow reserve and microvascular resistance predict increased hyperaemic coronary flow after elective percutaneous coronary intervention. Eur Heart J 2016; 37: 871.

23. Azzalini L, Candilio L, Poletti E, et al. Impact of post-percutaneous coronary intervention fractional flow reserve measurement on procedural management and clinical outcomes: The repeat-FFR study. J Am Coll Cardiol 2017; 70(18): B59.

24. Eid Madmani M, Abualsuod A, Edupuganti M, et al. Does post PCI FFR carry the same prognostic weight as non-ischemic FFR in deferred lesions? J Am Coll Cardiol 2017; 69(11): 1176.

25. Hakeem A, Uretsky BF. Clinical and prognostic value of post-stenting FFR. JACC Cardiovasc Interv 2017; 10(15): 1596.

26. Hoshino M, Yonetsu T, Murai T, et al. Determinants and clinical implication of post-procedural fractional flow reserve values as a predictor of major adverse cardiac events in patients with stable angina pectoris. Eur Heart J 2017; 38: 335.

27. Karamasis G, Mohdnazri SR, Al-Janabi F, et al. Fractional flow reserve post percutaneous coronary intervention for chronic total occlusions. Eur Heart J 2017; 38: 479.

28. Daemen J, Van Zandvoort L, Van Bommel R, et al. Explanation of post procedural fractional flow reserve below 0.85: A comprehensive ultrasound analysis of the ffr search registry. JACC Cardiovasc Interv 2018; 11(4): S36.

29. Hoshino M, Yonetsu T, Kanaji Y, et al. Determinants and clinical implication of post procedural fractional flow reserve as a predictor of major adverse cardiac events in patients. J Am Coll Cardiol 2018; 71(11).

30. Hoshino M, Yonetsu T, Kanaji Y, et al. Clinical significance of the fractional flow reserve measurement position after elective percutaneous coronary intervention. Eur Heart J 2018; 39: 952.

31. Jprn U. How does POst-PCI FFR value using pressure catheter relate to lesion prognosis or patient prognosis COmpaRed to aNatomical evaluation? http://www.who.int/trialsearch/Trial2aspx?TrialID=JPRN-UMIN000031963 2018.

32. Kobayashi Y, Fearon WF. Predicting outcomes after percutaneous coronary intervention using relative change in fractional flow reserve. JACC Cardiovasc Interv 2018; 11(20): 2110-2.

© 2022 Hwang D et al. *JAMA Network Open.*

33. Agarwal SK, Vallurupalli S, Siraj A, et al. Delineating the prognostic value of post PCI FFR in acute coronary syndrome patients. Circulation 2019; 140.

34. Hakeem A, Uretsky BF. Role of Postintervention fractional flow reserve to improve procedural and clinical outcomes. Circulation 2019; 139(5): 694-706.

35. Hakeem A, Uretsky BF. Toward a “More perfect” interventional algorithm: Post-intervention functional assessment using quantitative flow ratio. JACC Cardiovasc Interv 2019; 12(20): 2076-8.

36. Hamaya R, Kanaji Y, Usui E, et al. Improvement of fractional flow reserve after percutaneous coronary intervention does not necessarily indicate increased coronary flow. Eur Cardiol 2019; 14(1): 10-2.

37. Hoshino M, Kanaji Y, Sugano A, et al. Prognostic value of post intervention fractional flow reserve after intravascular ultrasound guided second generation drug eluting coronary stenting. J Am Coll Cardiol 2019; 73(9 Supplement 1): 1121.

38. Hou L, Kasula S, Kumar Agarwal S, et al. Post PCI hemodynamics: Predictive accuracy of post stenting Pd/Pa for determining post stenting ischemia. J J Am Coll Cardiol 2019; 73(9 Supplement 1): 1214.

39. Lim H, Yang HM, Yoon MH, et al. Usefulness of the trans-stent fractional flow reserve gradient for predicting clinical outcomes. Eur Heart J 2019; 40: 3726.

40. Madmani ME, Hasan R, Ayan M, et al. Comparison of prognostic value of fractional flow reserve in deferred patients with normal FFR with normal FFR post-PCI patients with stable coronary srtery disease. Catheter Cardiovasc Interv 2019; 93: S95-S6.

41. van Zandvoort LJC, Masdjedi K, Tovar Forero MN, et al. Fractional flow reserve guided percutaneous coronary intervention optimization directed by high-definition intravascular ultrasound versus standard of care: Rationale and study design of the prospective randomized FFR-REACT trial. Am Heart J 2019; 213: 66-72.

42. Van Zandvoort LJC, Masdjedi K, Witberg K, et al. Explanation of postprocedural fractional flow reserve below 0.85: A comprehensive ultrasound analysis of the FFR SEARCH registry. Circ Cardiovasc Interv 2019; 12(2): e007030.

43. Agarwal SK, Hakeem A, Hasan R, et al. Fractional flow reserve after functionally optimized coronary intervention (FCI) predicts long-term outcomes. J Am Coll Cardiol 2020; 75(11): 1456.

© 2022 Hwang D et al. *JAMA Network Open.*

eTable 1. List of Studies Met the Criteria for the Post-PCI FLOW Registry

No.	Title	First Author	Journal	Provided raw data
1	The impact of fractional flow reserve measurement on clinical outcomes after transradial coronary stenting.	Leesar, M. A., et al.	EuroIntervention (2011)[1]	Yes
2	Relation of fractional flow reserve after drug-eluting stent implantation to one-year outcomes.	Nam, C. W., et al.	Am J Cardiol (2011)[2]	Yes
3	Hemodynamic changes of fractional flow reserve after double kissing crush and provisional stenting technique for true bifurcation lesions.	Ye, F., et al.	Chin Med J (Engl) (2012)[3]	Yes
4	Clinical implications of coronary pressure measurement after stent implantation.	Matsuo, A., et al.	Cardiovasc Interv Ther (2013)[4]	Yes
5	Relationship between fractional flow reserve and residual plaque volume and clinical outcomes after optimal drug-eluting stent implantation: insight from intravascular ultrasound volumetric analysis	Ito, T., et al.	Int J Cardiol (2014)[5]	Yes
6	Prognostic value of fractional flow reserve: linking physiologic severity to clinical outcomes.	Johnson, N. P., et al.	J Am Coll Cardiol (2014)[6]	Yes
7	Clinical Relevance of Poststent Fractional Flow Reserve After Drug-Eluting Stent Implantation.	Doh, J. H., et al.	J Invasive Cardiol (2015)[7]	Yes
8	Correlation between OCT-derived intrastent dimensions and fractional flow reserve measurements after coronary stent implantation and impact on clinical outcome.	Reith, S., et al.	J Invasive Cardiol (2015)[8]	No
9	Utilizing Post-Intervention Fractional Flow Reserve to Optimize Acute Results and the Relationship to Long-Term Outcomes.	Agarwal, S. K., et al.	JACC Cardiovasc Interv (2016)[9]	Yes
10	Clinical and prognostic value of poststenting fractional flow reserve in acute coronary syndromes.	Kasula, S., et al.	Heart (2016)[10]	Yes
11	Prevalence and Clinical Significance of Discordant Changes in Fractional and Coronary Flow Reserve After Elective Percutaneous Coronary Intervention.	Matsuda, J., et al.	J Am Heart Assoc (2016)[11]	Yes

© 2022 Hwang D et al. *JAMA Network Open.*

12	Cutoff Value and Long-Term Prediction of Clinical Events by FFR Measured Immediately After Implantation of a Drug-Eluting Stent in Patients With Coronary Artery Disease: 1- to 3-Year Results From the DKCRUSH VII Registry Study.	Li, S. J., et al.	JACC Cardiovasc Interv (2017)[12]	Yes
13	Prognostic Value of Fractional Flow Reserve Measured Immediately After Drug-Eluting Stent Implantation.	Piroth, Z., et al.	Circ Cardiovasc Interv (2017)[13]	Yes
14	Prognostic Implications of Relative Increase and Final Fractional Flow Reserve in Patients With Stent Implantation.	Lee, J. M., et al.	JACC Cardiovasc Interv (2018)[14]	Yes
15	Clinical significance of concordance or discordance between fractional flow reserve and coronary flow reserve for coronary physiological indices, microvascular resistance, and prognosis after elective percutaneous coronary intervention.	Usui, E., et al.	EuroIntervention (2018)[15]	Yes
16	Impact of Post-Percutaneous Coronary Intervention Fractional Flow Reserve Measurement on Procedural Management and Clinical Outcomes: The REPEAT-FFR Study.	Azzalini, L., et al.	J Invasive Cardiol (2019)[16]	Yes
17	Incremental Prognostic Value of Post-Intervention Pd/Pa in Patients Undergoing Ischemia-Driven Percutaneous Coronary Intervention.	Hakeem, A., et al.	JACC Cardiovasc Interv (2019)[17]	Yes
18	Prognostic value of post-intervention fractional flow reserve after intravascular ultrasound-guided second-generation drug-eluting coronary stenting.	Hoshino, M., et al.	EuroIntervention (2019)[18]	Yes
19	Influence of target vessel on prognostic relevance of fractional flow reserve after coronary stenting.	Hwang, D., et al.	EuroIntervention (2019)[19]	Yes
20	Usefulness of the trans-stent fractional flow reserve gradient for predicting clinical outcomes.	Yang, H. M., et al.	Catheter Cardiovasc Interv (2019)[20]	Yes
21	Role of Post-Stent Physiological Assessment in a Risk Prediction Model After Coronary Stent Implantation.	Hwang, D., et al.	JACC Cardiovasc Interv (2020)[21]	Yes
22	Prognostic Value of Prerevascularization Fractional FlowReserve Mediated by the Postrevascularization Level	Hamaya, R.,et al.	JAMA Network Open (2020)[22]	Yes

© 2022 Hwang D et al. *JAMA Network Open.*

23	Prognostic Implications of Post-Intervention Resting Pd/Pa and Fractional Flow Reserve in Patients With Stent Implantation.	Shin, D., et al.	JACC Cardiovasc Interv (2020)[23]	Yes
24	Insufficient recovery of fractional flow reserve even after optimal implantation of drug-eluting stents: 3-year outcomes from the FUJI study.	Hokama, Y., et al.	J Cardiol (2020)[24]	Yes
25	Physiological Distribution and Local Severity of Coronary Artery Disease and Outcomes After Percutaneous Coronary Intervention.	Shin, D., et al.	JACC Cardiovasc Interv (2021)[25]	Yes
26	Effect of Coronary Disease Characteristics on Prognostic Relevance of Residual Ischemia After Stent Implantation.	Yang, S., et al.	Front Cardiovasc Med (2021)[26]	Yes
27	Differential Prognostic Implications of Pre- and Post-Stent Fractional Flow Reserve in Patients Undergoing Percutaneous Coronary Intervention.	Zhang, J., et al.	Korean Circ J (2022)[27]	Yes
28	Impact of Poststenting Fractional Flow Reserve on Long-Term Clinical Outcomes	Diletti, R., et al.	Circ Cardiovasc Interv (2021)[28]	Yes
29	Post-stenting fractional flow reserve vs coronary angiography for optimization of percutaneous coronary intervention (TARGET-FFR).	Collison, D., et al.	Eur Heart J (2021)[29]	Yes

© 2022 Hwang D et al. *JAMA Network Open.*

eTable 2. List of Studies and Cohorts Included in the Post-PCI FLOW Registry

No.	Title	First Author	Cohort
1	The impact of fractional flow reserve measurement on clinical outcomes after transradial coronary stenting.	Leesar, M. A., et al.	Institutional cohort from affiliated center
2	Relation of fractional flow reserve after drug-eluting stent implantation to one-year outcomes.	Nam, C. W., et al.	Institutional cohort from affiliated center
3	Hemodynamic changes of fractional flow reserve after double kissing crush and provisional stenting technique for true bifurcation lesions.	Ye, F., et al.	Institutional cohort from affiliated center
4	Clinical implications of coronary pressure measurement after stent implantation.	Matsuo, A., et al.	Institutional cohort from affiliated center
5	Relationship between fractional flow reserve and residual plaque volume and clinical outcomes after optimal drug-eluting stent implantation: insight from intravascular ultrasound volumetric analysis	Ito, T., et al.	Institutional cohort from affiliated center
6	Prognostic value of fractional flow reserve: linking physiologic severity to clinical outcomes.	Johnson, N. P., et al.	Meta-analysis
7	Clinical Relevance of Poststent Fractional Flow Reserve After Drug-Eluting Stent Implantation.	Doh, J. H., et al.	Institutional cohort from affiliated center
8	Utilizing Post-Intervention Fractional Flow Reserve to Optimize Acute Results and the Relationship to Long-Term Outcomes.	Agarwal, S. K., et al.	Central Arkansas VA Health systems
9	Clinical and prognostic value of poststenting fractional flow reserve in acute coronary syndromes.	Kasula, S., et al.	Central Arkansas VA Health systems
10	Prevalence and Clinical Significance of Discordant Changes in Fractional and Coronary Flow Reserve After Elective Percutaneous Coronary Intervention.	Matsuda, J., et al.	Institutional cohort from affiliated center
11	Cutoff Value and Long-Term Prediction of Clinical Events by FFR Measured Immediately After Implantation of a Drug-Eluting Stent in Patients With Coronary Artery Disease: 1- to 3-Year Results From the DKCRUSH VII Registry Study.	Li, S. J., et al.	DKCRUSH VII

© 2022 Hwang D et al. *JAMA Network Open.*

12	Prognostic Value of Fractional Flow Reserve Measured Immediately After Drug-Eluting Stent Implantation.	Piroth, Z., et al.	FAME 1 and FAME 2
13	Prognostic Implications of Relative Increase and Final Fractional Flow Reserve in Patients With Stent Implantation.	Lee, J. M., et al.	COE PERSPECTIVE
14	Clinical significance of concordance or discordance between fractional flow reserve and coronary flow reserve for coronary physiological indices, microvascular resistance, and prognosis after elective percutaneous coronary intervention.	Usui, E., et al.	Institutional cohort from affiliated center
15	Impact of Post-Percutaneous Coronary Intervention Fractional Flow Reserve Measurement on Procedural Management and Clinical Outcomes: The REPEAT-FFR Study.	Azzalini, L., et al.	Institutional cohort from affiliated center
16	Incremental Prognostic Value of Post-Intervention Pd/Pa in Patients Undergoing Ischemia-Driven Percutaneous Coronary Intervention.	Hakeem, A., et al.	Central Arkansas VA Health systems
17	Prognostic value of post-intervention fractional flow reserve after intravascular ultrasound-guided second-generation drug-eluting coronary stenting.	Hoshino, M., et al.	Institutional cohort from affiliated center
18	Influence of target vessel on prognostic relevance of fractional flow reserve after coronary stenting.	Hwang, D., et al.	COE PERSPECTIVE
19	Usefulness of the trans-stent fractional flow reserve gradient for predicting clinical outcomes.	Yan-g, H. M., et al.	Institutional cohort from affiliated center
20	Role of Post-Stent Physiological Assessment in a Risk Prediction Model After Coronary Stent Implantation.	Hwang, D., et al.	International Post-PCI FFR registry (DKCRUSH VII, COE PERSPECTIVE, 3V FFR FRIENDS, Institutional cohort from Tsuchiura Kyodo General Hospital)
21	Prognostic Value of Prerevascularization Fractional FlowReserve Mediated by the Postrevascularization Level	Hamaya, R.,et al.	International Post-PCI FFR registry (DKCRUSH VII, COE PERSPECTIVE, 3V FFR FRIENDS, Institutional cohort from Tsuchiura Kyodo General Hospital)
22	Prognostic Implications of Post-Intervention Resting Pd/Pa and Fractional Flow Reserve in Patients With Stent Implantation.	Shin, D., et al.	PERSPECTIVE PCI

© 2022 Hwang D et al. *JAMA Network Open.*

23	Insufficient recovery of fractional flow reserve even after optimal implantation of drug-eluting stents: 3-year outcomes from the FUJI study.	Hokama, Y., et al.	FUJI study
24	Physiological Distribution and Local Severity of Coronary Artery Disease and Outcomes After Percutaneous Coronary Intervention.	Shin, D., et al.	PERSPECTIVE PCI
25	Effect of Coronary Disease Characteristics on Prognostic Relevance of Residual Ischemia After Stent Implantation.	Yang, S., et al.	International Post-PCI FFR registry (DKCRUSH VII, COE PERSPECTIVE, 3V FFR FRIENDS, Institutional cohort from Tsuchiura Kyodo General Hospital)
26	Differential Prognostic Implications of Pre- and Post-Stent Fractional Flow Reserve in Patients Undergoing Percutaneous Coronary Intervention.	Zhang, J., et al.	International Post-PCI FFR registry (DKCRUSH VII, COE PERSPECTIVE, 3V FFR FRIENDS, Institutional cohort from Tsuchiura Kyodo General Hospital)
27	Impact of Poststenting Fractional Flow Reserve on Long-Term Clinical Outcomes	Diletti, R., et al.	FFR-SEARCH
28	Post-stenting fractional flow reserve vs coronary angiography for optimization of percutaneous coronary intervention (TARGET-FFR).	Collison, D., et al.	TARGET-FFR

© 2022 Hwang D et al. *JAMA Network Open.*

eTable 3. Number of Patients Provided by Each Cohort and Number of Patients Included in the Master Data Set

No.	Study	Provided patient number	Included patient number	Specific comments
1	FAME 1 and FAME 2	639	639	Full data was available.
2	Central Arkansas VA Health systems	574	450	Exclude patients after PTCA and BMS
3	DKCRUSH VII	780	774	Only data from the centers that agreed to provide it were collected.
4	COE PERSPECTIVE	835	822	Excluded patients with missing data
5	3V FFR FRIENDS	266	258	Patients with post-PCI FFR data were included. Excluded patients with missing data
6	Kakuta, et al.	347	346	Excluded patients with missing data
7	PERSPECTIVE PCI	268	268	PERSPECTIVE PCI has overlapped the study population with the COE-PERSPECTIVE registry. Half of the data was from Samsung Medical center.
8	FUJI study	218	218	Full data was available
9	Johnson, N. P., et al.	966	152	Patients after PTCA or BMS implantation were excluded.<br>Patients without clear outcome data were excluded.
10	Leesar, M. A., et al.	66	0	There were no data for stent information.
11	Matsuo, A., et al.	100	65	Patients after PTCA or BMS implantation were excluded.<br>Patients without clear outcome data were excluded.
12	Ito, T., et al.	97	97	Full data was available.
13	Doh, J. H., et al.	107	107	Full data was available.

© 2022 Hwang D et al. *JAMA Network Open.*

14	Azzalini, L., et al.	65	58	Patients after DEB or BRS implantation were excluded.
15	Yang, H. M., et al.	135	135	Full data was available.
16	FFR-SEARCH	959	628	Exclude patients with ST-elevation myocardial infarction or with graft vessel post-PCI FFR
17	TARGET-FFR	260	260	Full data was available.

Abbreviations: BMS, bare-metal stent; BRS, bioresorbable scaffold; FFR, fractional flow reserve; PCI, percutaneous coronary intervention; PTCA, percutaneous transluminal coronary angioplasty.

© 2022 Hwang D et al. *JAMA Network Open.*

eTable 4. Description of the Included Cohorts

No.	Cohort	Study type	Time perspective	Recruitment center	Study population	Follow-up duration	Stent type	Angiographic successful PCI
1	FAME 1 and FAME 2	Observational cohort from RCTs	prospective	FAME 1 from 20 centers and FAME 2 form 28 centers	FAME 1 (352): Angiographic multivessel CAD + FFR-guided arm<br>FAME 2 (287): SIHD or stabilized ACS with 1, 2, or 3 VD.	2 years	DES	Yes
2	Central Arkansas VA Health systems	Observational cohort	retrospective	1 center	390 SIHD or 184 ACS patients	31 months	BMS/DES	Yes
3	DKCRUSH VII	Observational cohort	prospective	9 centers	1,496 patients	3 years	DES	Yes
4	COE PERSPECTIVE	Observational cohort	prospective	9 centers	452 SIHD patients, 383 ACS patients	2 years	DES	Yes
5	3V FFR FRIENDS	Observational cohort	prospective	4 centers	882 SIHD patients, 254 ACS patients	2 years	DES	Yes
6	Kakuta, et al.	Observational cohort	prospective	1 center	311 SIHD patients, 35 ACS patients	2 years	DES	Yes
7	PERSPECTIVE PCI	Observational cohort	prospective	5 centers	309 SIHD patients, 279 ACS patients	2 years	DES	Yes
8	FUJI study	Observational cohort	prospective	17 centers	200 SIHD patients, 18 UA patients	31.4 months	DES	Yes
9	Johnson, N. P., et al.	Meta-analysis	retrospective	From 15 studies	966 patients	Median 12 months-	BMS/DES	NA
10	Leesar, M. A., et al.	Observational cohort	prospective	1 center	66 SIHD patients	2 years	BMS/DES	Yes
11	Matsuo, A., et al.	Observational cohort	prospective	1 center	69 patients	6 months	BMS/DES	Yes

© 2022 Hwang D et al. *JAMA Network Open.*

12	Ito, T., et al.	Observational cohort	retrospective	1 center	89 SIHD patients, 8 UA patients	17.8 months	DES	Yes
13	Doh, J. H., et al.	Observational cohort	prospective	1 center	72 SIHD patients, 35 ACS patients	3 years	DES	Yes
14	Azzalini, L., et al.	Observational cohort	prospective	1 center	50 SIHD patients, 15 ACS patients	1 year	DES/BRS/DEB	Yes
15	Yang, H. M., et al.	Observational cohort	retrospective	1 center	57 SIHD patients, 78 UA patients	6 years	DES	Yes
16	FFR-SEARCH	Observational cohort	prospective	1 center	284 SIHD patients, 344 ACS patients	2 years	DES	Yes
17	TARGET-FFR	Randomized controlled trial	prospective	1 center	72 SIHD patients, 188 ACS patients	2 years	DES	Yes

Abbreviations: ACS, acute coronary syndrome; BMS, bare-metal stent; BRS, bioresorbable scaffold; CAD, coronary artery disease; DEB, drug-eluting balloon; DES, drug-eluting stent; FFR, fractional flow reserve; NA, not available; PCI, percutaneous coronary intervention; RCT, randomized controlled trial; SIHD, stable ischemic heart disease; UA, unstable angina; VD, vessel disease.

© 2022 Hwang D et al. *JAMA Network Open.*

eTable 5. The Newcastle-Ottawa Scale for Assessing the Quality of Observational Cohorts

Cohort	Selection*	Comparability†	Outcome‡	Quality
FAME 1 and FAME 2	★★★	★★	★★★	8/8
Central Arkansas VA Health systems	★★★	★	★★★	7/8
DKCRUSH VII	★★★	★★	★★★	8/8
COE PERSPECTIVE	★★★	★★	★★★	8/8
3V FFR FRIENDS	★★★	★★	★★★	8/8
Kakuta et al.	★★★	★★	★★★	8/8
PERSPECTIVE PCI	★★★	★★	★★★	8/8
FUJI study	★★★	★★	★★★	8/8
Johnson, N. P., et al.	★★		★★	4/8
Leesar, M. A., et al.	★★	★	★★	5/8
Matsuo, A., et al.	★★	★★	★★	6/8
Ito, T., et al.	★★★	★	★★★	7/8
Doh, J. H., et al.	★★★	★★	★★★	8/8
Azzalini, L., et al.	★★★	★★	★★	7/8
Yang, H. M., et al.	★★	★	★★★	6/8
FFR-SEARCH	★★★	★★	★★★	8/8
TARGET-FFR	★★★	★★	★★★	8/8

*Selection process was assessed with representativeness of the cohort, ascertainment of exposure of the cohort and presence of all interested outcomes.

†Comparability was assessed with design of cohort (prospective/retrospective) and availability of risk factors.

‡Outcome was assessed with whether outcomes were clearly provided, duration of follow-up and completeness of follow-up.

The quality of each study was assessed based on a Newcastle-Ottawa scale. We assessed each study's selection process, comparability, and outcome for a maximum total of 8 points (3 points for selection, 2 points for comparability, and 3 points for outcome). Studies were ranked high if they had a score of >5, moderate if they had a score of 3 to 5, and low if they had a score of <3.

© 2022 Hwang D et al. *JAMA Network Open.*

eTable 6. Baseline Characteristics of the Study Population

**General characteristics**	
Age, years	64.4± 10.1
Male	4,141 (78.5%)
**Cardiovascular risk factors**	
Hypertension	3,628 (68.8%)
Diabetes mellitus	1,799 (34.1%)
Hypercholesterolemia	3,140 (59.6%)
Current smoker	1,687 (32.0%)
Previous MI	1,020 (20.4%)
**Clinical presentations**	
Acute coronary syndrome	2,064 (39.5%)
Stable coronary artery disease	3,156 (60.5%)
**Target vessel**	
LAD	3,565 (67.8%)
LCX	691 (13.1%)
RCA	999 (19.0%)
**Pre-PCI FFR**	0.71 (0.61-0.77)
**Post-PCI FFR**	0.88 (0.84-0.93)

Values are mean ± standard deviation, median (interquartile ranges, 25th-75th), or n (%).

Abbreviations: FFR, fractional flow reserve; LAD, left anterior descending artery; LCX, left circumflex artery; MI, myocardial infarction; PCI, percutaneous coronary intervention; RCA, right coronary artery.

© 2022 Hwang D et al. *JAMA Network Open.*

eTable 7. Per-vessel Specific Characteristics and Outcomes

**Vessel characteristics**	
**Total number of vessels**	5,869
**Vessel location**	
LAD	3,697 (63.2%)
LCX	927 (15.9%)
RCA	1,223 (20.9%)
**Pre-PCI FFR**	0.71 (0.61-0.77)
**Post-PCI FFR**	0.89 (0.84-0.94)
**Vessel-specific outcome**	
Target vessel myocardial infarction	63/5,796 (1.2%)*
Target vessel revascularization	309/5,868 (5.9%)*

*The cumulative incidence of clinical outcomes at 2 years is presented as Kaplan-Meier estimates.

Abbreviations: FFR, fractional flow reserve; LAD, left anterior descending artery; LCX, left circumflex artery; PCI, percutaneous coronary intervention; RCA, right coronary artery.

© 2022 Hwang D et al. *JAMA Network Open.*

eTable 8. Cumulative Incidence and Risk of Clinical Events According to Post-PCI FFR Strata

	**Event (%)***	**HR (95% CI)**	**p-value**	**Adjusted HR (95% CI)** †	**p-value**
TVF					
**0.95<Post-PCI FFR (n=745)**	38/737 (5.5%)	Reference			
**0.90<Post-PCI FFR≤0.95 (n=1,290)**	69/1,272 (6.1%)	1.080 (0.726-1.606)	0.70	1.052 (0.706-1.569)	0.80
**0.85<Post-PCI FFR≤0.90 (n=1,470)**	93/1,451 (7.1%)	1.329 (0.910-1.941)	0.14	1.333 (0.911-1.950)	0.14
**0.80<Post-PCI FFR≤0.85 (n=1,095)**	80/1,075 (8.3%)	1.649 (1.118-2.432)	0.01	1.604 (1.081-2.381)	0.02
**Post-PCI FFR≤0.80 (n=677)**	60/669 (10.1%)	2.106 (1.397-3.176)	<0.001	2.108 (1.385-3.209)	<0.001
Cardiac death or TVMI					
**0.95<Post-PCI FFR (n=745)**	13/737 (1.9%)	Reference			
**0.90<Post-PCI FFR≤0.95 (n=1,290)**	25/1,272 (2.2%)	1.251(0.634-2.471)	0.52	1.255 (0.633-2.490)	0.52
**0.85<Post-PCI FFR≤0.90 (n=1,470)**	29/1,451 (2.2%)	1.353 (0.682-2.684)	0.39	1.356 (0.681-2.700)	0.39
**0.80<Post-PCI FFR≤0.85 (n=1,095)**	23/1,075 (2.4%)	1.646 (0.783-3.460)	0.19	1.636 (0.772-3.467)	0.20
**Post-PCI FFR≤0.80 (n=677)**	21/669 (3.6%)	2.590 (1.141-5.880)	0.02	2.559 (1.116-5.867)	0.03

*The cumulative incidence of clinical outcomes at 2 years is presented as Kaplan-Meier estimates.

†The following patient risk factors were included in the multivariable-adjusted mixed-effects Cox proportional hazard regression model: age, sex, hypertension, diabetes mellitus, hypercholesterolemia, and acute coronary syndrome.

Abbreviations: CI, confidence interval; FFR, fractional flow reserve; HR, hazard ratio; PCI, percutaneous coronary intervention; TVF, target vessel failure; TVMI, target vessel myocardial infarction.

© 2022 Hwang D et al. *JAMA Network Open.*

eTable 9. The Risk of Clinical Events at 2 Years per Post-PCI FFR 0.01 Decrease in Subgroups

	Patient number	Event (%)*	HR (95% CI)	p-value	Adjusted HR (95% CI) †	p-value	Interaction p-value
TVF							
**Age≥65 years**	2,656	153 (8.0%)	1.026 (0.996-1.056)	0.09	1.024 (0.995-1.054)	0.09	0.92
**Age<65years**	2,620	187 (6.5%)	1.032 (1.009-1.054)	0.005	1.034 (1.010-1.059)	0.005	
**Male**	4,141	267 (7.3%)	1.027 (1.008-1.047)	0.006	1.027 (1.005-1.050)	0.02	0.55
**Female**	1,135	73 (7.1%)	1.061 (1.021-1.102)	0.002	1.045 (1.009-1.081)	0.01	
**With HTN**	3,628	261 (8.1%)	1.038 (1.021-1.056)	<0.001	1.040 (1.022-1.058)	<0.001	0.51
**Without HTN**	1,643	79 (5.3%)	1.021 (0.991-1.051)	0.17	1.016 (0.981-1.054)	0.37	
**With DM**	1,799	144 (9.3%)	1.017 (0.986-1.049)	0.27	1.015 (0.982-1.049)	0.38	0.86
**Without DM**	3,475	196 (6.2%)	1.032 (1.011-1.054)	0.003	1.033 (1.011-1.055)	0.003	
**ACS**	2,064	141 (7.4%)	1.031 (1.006-1.056)	0.01	1.029 (1.004-1.055)	0.02	0.40
**Non-ACS**	3,156	195 (7.1%)	1.037 (1.017-1.058)	<0.001	1.039 (1.018-1.061)	<0.001	
Cardiac death or TVMI							
**Age≥65 years**	2,656	67 (2.9%)	1.034 (0.989-1.080)	0.14	1.032 (0.987-1.080)	0.16	0.47
**Age<65 years**	2,620	44 (1.9%)	1.026 (0.982-1.071)	0.26	1.025 (0.980-1.071)	0.28	
**Male**	4,141	89 (2.5%)	1.029 (0.993-1.067)	0.11	1.030 (0.992-1.068)	0.12	0.34
**Female**	1,135	22 (2.1%)	1.070 (1.009-1.134)	0.02	1.056 (0.994-1.122)	0.08	
**With HTN**	3,628	85 (2.7%)	1.029 (0.995-1.064)	0.10	1.029 (0.994-1.066)	0.11	0.68
**Without HTN**	1,643	26 (1.8%)	1.046 (0.987-1.108)	0.13	1.049 (0.987-1.114)	0.12	
**With DM**	1,799	53 (3.4%)	1.007 (0.963-1.053)	0.76	1.012 (0.967-1.060)	0.60	0.31
**Without DM**	3,475	58 (1.9%)	1.050 (1.010-1.092)	0.02	1.048 (1.007-1.091)	0.02	
**ACS**	2,064	48 (2.6%)	1.038 (0.982-1.098)	0.18	1.031 (0.976-1.090)	0.27	0.50
**Non-ACS**	3,156	63 (2.3%)	1.017 (0.981-1.054)	0.36	1.018 (0.982-1.056)	0.33	

*The cumulative incidence of clinical outcomes at 2 years is presented as Kaplan-Meier estimates.

†The following patient risk factors were included in the multivariable-adjusted mixed-effects Cox proportional hazard regression model: age, sex, hypertension, diabetes mellitus, hypercholesterolemia, and acute coronary syndrome.

Abbreviations: ACS, acute coronary intervention; CI, confidence interval; DM, diabetes mellitus; FFR, fractional flow reserve; HR, hazard ratio; HTN, hypertension; PCI, percutaneous coronary intervention; TVF, target vessel failure; TVMI, target vessel myocardial infarction.

© 2022 Hwang D et al. *JAMA Network Open.*

eTable 10. Predictors of TVF and Cardiac Death or TVMI

Variables	Adjusted HR*	95% CI	p-value
**TVF**			
**Age, per 1 year increase**	1.012	1.001-1.024	0.03
**Male**	1.013	0.772-1.328	0.93
**Hypertension**	1.317	1.006-1.725	0.04
**Diabetes mellitus**	1.356	1.083-1.699	0.008
**Hypercholesterolemia**	0.996	0.786-1.262	0.97
**Acute coronary syndrome**	1.360	1.065-1.736	0.01
**Post-PCI FFR, per 0.01 decrease**	1.035	1.020-1.051	<0.001
**Cardiac death or TVMI**			
**Age, per 1 year increase**	1.038	1.017-1.060	<0.001
**Male**	1.171	0.720-1.905	0.52
**Hypertension**	1.151	0.718-1.845	0.56
**Diabetes mellitus**	1.814	1.225-2.687	0.003
**Hypercholesterolemia**	0.981	0.646-1.491	0.93
**Acute coronary syndrome**	1.680	1.094-2.580	0.02
**Post-PCI FFR, per 0.01 decrease**	1.034	1.001-1.068	0.049

*The following patient risk factors were included in the multivariable-adjusted mixed-effects Cox proportional hazard regression model: age, sex, hypertension, diabetes mellitus, hypercholesterolemia, and acute coronary syndrome.

Abbreviations: CI, confidence interval; FFR, fractional flow reserve; HR, hazard ratio; PCI, percutaneous coronary intervention; TVF, target vessel failure; TVMI, target vessel myocardial infarction.

© 2022 Hwang D et al. *JAMA Network Open.*

eTable 11. Clinical Events According to Post-PCI FFR Cut-off Value

	Post-PCI FFR		HR (95% CI)	p-value	Adjusted HR* (95% CI)	p-value
	**≤0.86**	**>0.86**				
**TVF**	165/2,021 (9.1%)	175/3,183 (6.1%)	1.658 (1.334-2.062)	<0.001	1.575 (1.241-1.999)	<0.001
**Cardiac death or TVMI**	52/2,021 (2.9%)	59/3,183 (2.1%)	1.649 (1.109-2.452)	0.01	1.562 (1.035-2.355)	0.03
Cardiac death	33/2,055 (1.9%)	31/3,219 (1.1%)	1.829 (1.107-3.020)	0.02	1.748 (1.058-2.890)	0.03
TVMI	23/2,023 (1.2%)	34/3,184(1.2%)	1.450 (0.782-2.686)	0.24	1.425 (0.767-2.646)	0.26
TVR	140/2,056 (7.7%)	145/3,220 (5.0%)	1.655 (1.294-2.119)	<0.001	1.619 (1.267-2.067)	<0.001
	**Post-PCI FFR**		**HR (95% CI)**	**p-value**	**Adjusted HR* (95% CI)**	**p-value**
	**≤0.80**	**>0.80**				
**TVF**	60/669 (10.1%)	280/4,535 (6.8%)	1.524 (1.123-2.069)	0.007	1.509 (1.102-2.067)	0.01
**Cardiac death or TVMI**	21/669 (3.6%)	90/4,535 (2.2%)	1.820 (1.079-3.068)	0.03	1.821 (1.080-3.072)	0.03
Cardiac death	12/677(2.1%)	52/4,597 (1.3%)	1.610 (0.834-3.107)	0.16	1.668 (0.876-3.176)	0.12
TVMI	10/669 (1.7%)	47/4,538 (1.1%)	2.025 (0.959-4.276)	0.06	2.103 (0.996-4.441)	0.05
TVR	51/677 (8.5%)	234/4,599 (5.7%)	1.629 (1.192-2.226)	0.002	1.579 (1.132-2.204)	0.007

*The following patient risk factors were included in the multivariable-adjusted mixed-effects Cox proportional hazard regression model: age, sex, hypertension, diabetes mellitus, hypercholesterolemia, and acute coronary syndrome.

Abbreviations: CI, confidence interval; FFR, fractional flow reserve; HR, hazard ratio; PCI, percutaneous coronary intervention; TVF, target vessel failure; TVMI, target vessel myocardial infarction; TVR, target vessel revascularization.

© 2022 Hwang D et al. *JAMA Network Open.*

eFigure 1. Flow chart of study selection process

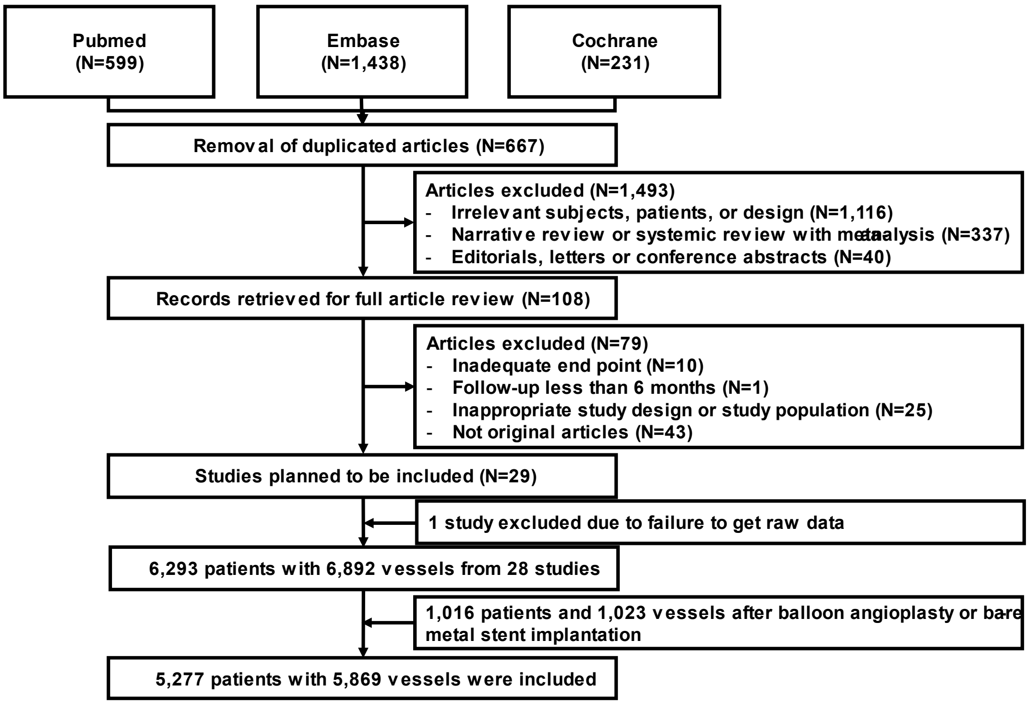

Study flow chart following the guideline of PRISMA-IPD (Preferred Reporting Items for Systematic Review and Meta-analysis of Individual Participant Data).

© 2022 Hwang D et al. *JAMA Network Open.*

eFigure 2. Hazard ratios of clinical events according to postPCI FFR strata

A. TVF

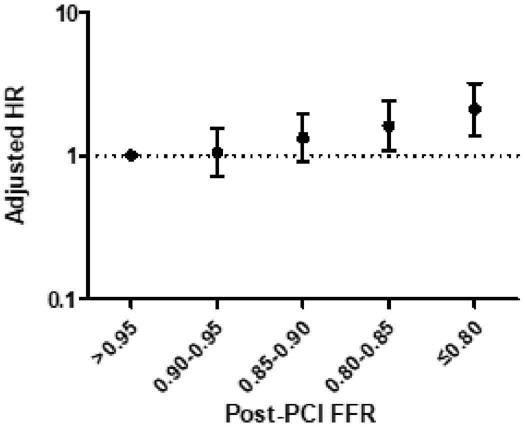

B. Cardiac death or TVMI

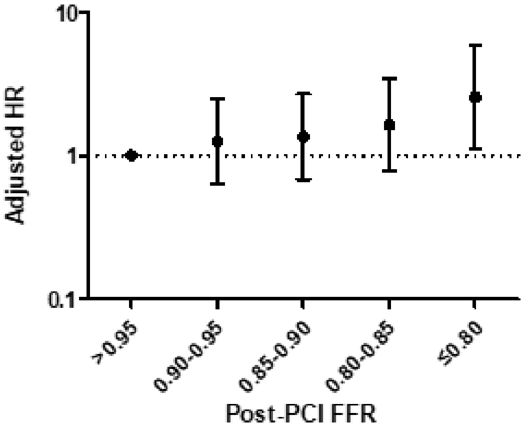

With the patients with post-PCI FFR over 0.95 as a reference, the risks of TVF (A) and cardiac death or TVMI (B) at 2 years are presented. The risks are presented as adjusted HR by multivariable-adjusted mixed-effects Cox proportional hazard model.

Abbreviations: FFR, fractional flow reserve; HR, hazard ratio; PCI, percutaneous coronary intervention; TVF, target vessel failure; TVMI, target vessel myocardial infarction.

© 2022 Hwang D et al. *JAMA Network Open.*

eFigure 3. The risks of clinical events per post-PCI FFR 0.01 decrease according to subgroups

	Patient number	Adjusted HR* (95% CI)	p-value	Interaction p-value
TVF				
Age≥ 65 years	2,656	1.024 (0.995 -1.054)	0.09	0.92
Age< 65years	2,620	1.034 (1.010 -1.059)	0.005	
Male	4,141	1.027 (1.005 -1.050)	0.02	0.55
Female	1,135	1.045 (1.009 -1.081)	0.01	
With HTN	3,628	1.040 (1.022 -1.058)	<0.001	0.51
Without HTN	1,643	1.016 (0.981 -1.054)	0.37	
With DM	1,799	1.015 (0.982 -1.049)	0.38	0.86
Without DM	3,475	1.033 (1.011 -1.055)	0.003	
ACS	2,064	1.029 (1.004 -1.055)	0.02	0.40
Non-ACS	3,156	1.039 (1.018 -1.061)	<0.001	
Cardiac death or TVMI				
Age≥ 65 years	2,656	1.032 (0.987 -1.080)	0.16	0.47
Age< 65years	2,620	1.025 (0.980 -1.071)	0.28	
Male	4,141	1.030 (0.992 -1.068)	0.12	0.34
Female	1,135	1.056 (0.994 -1.122)	0.08	
With HTN	3,628	1.029 (0.994 -1.066)	0.11	0.68
Without HTN	1,643	1.049 (0.987 -1.114)	0.12	
With DM	1,799	1.012 (0.967 -1.060)	0.60	0.31
Without DM	3,475	1.048 (1.007 -1.091)	0.02	
ACS	2,064	1.031 (0.976 -1.090)	0.27	0.50
Non-ACS	3,156	1.018 (0.982 -1.056)	0.33	

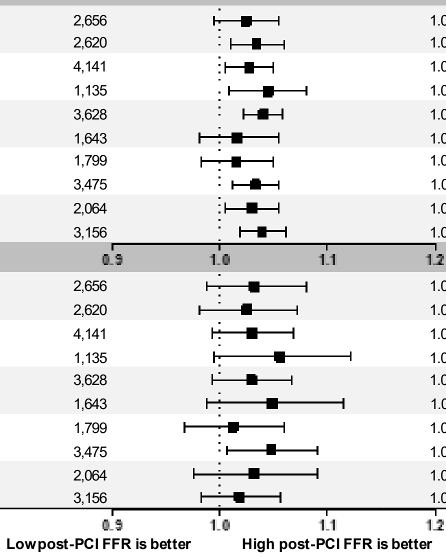

Prespecified subgroup analyses for age, sex, hypertension, diabetes mellitus, and clinical diagnosis show consistent results with the main findings.

Abbreviations: ACS, acute coronary syndrome; CI, confidence interval; DM, diabetes mellitus; FFR, fractional flow reserve; HR, hazard ratio; HTN, hypertension; PCI, percutaneous coronary intervention; TVF, target vessel failure; TVMI, target vessel myocardial infarction.

© 2022 Hwang D et al. *JAMA Network Open.*

eFigure 4. Optimal cut-off values of post-PCI FFR for predicting future events

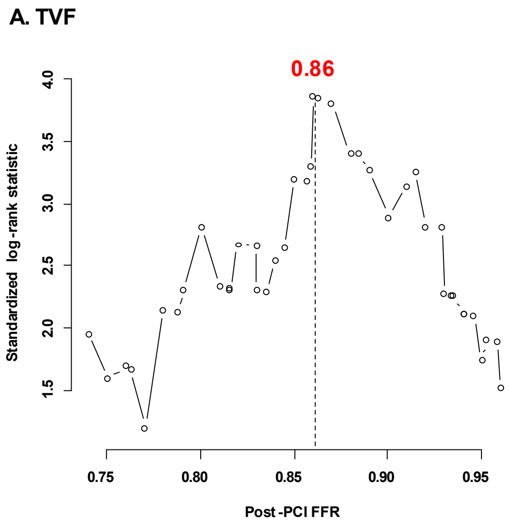

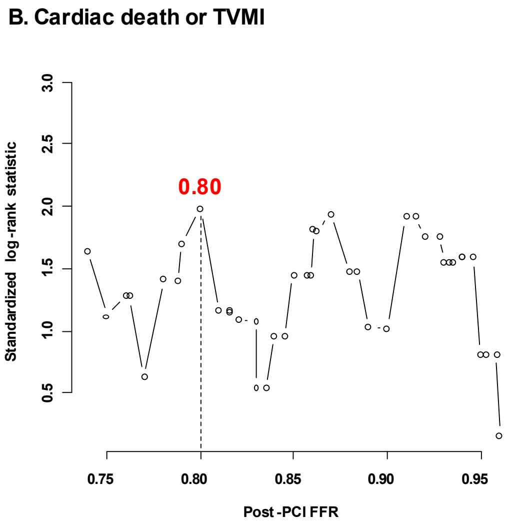

The optimal cut-off values of post-PCI FFR were calculated based on maximizing the difference of log-rank statistics for TVF and cardiac death or TVMI at 2 years.

Abbreviations: FFR, fractional flow reserve; PCI, percutaneous coronary intervention; TVF, target vessel failure; TVMI, target vessel myocardial infarction.

© 2022 Hwang D et al. *JAMA Network Open.*

eReferences

1. Leesar MA, Satran A, Yalamanchili V, Helmy T, Abdul-Waheed M and Wongpraparut N. The impact of fractional flow reserve measurement on clinical outcomes after transradial coronary stenting. *EuroIntervention*. 2011;7:917-23.
2. Nam CW, Hur SH, Cho YK, Park HS, Yoon HJ, Kim H, Chung IS, Kim YN, Kim KB, Doh JH, Koo BK, Tahk SJ and Fearon WF. Relation of fractional flow reserve after drug-eluting stent implantation to one-year outcomes. *Am J Cardiol*. 2011;107:1763-7.
3. Ye F, Chen SL, Zhang JJ, Zhu ZS, Kan J, Tian NL, Lin S, Liu ZZ, You W, Xu HM and Xu J. Hemodynamic changes of fractional flow reserve after double kissing crush and provisional stenting technique for true bifurcation lesions. *Chin Med J (Engl)*. 2012;125:2658-62.
4. Matsuo A, Fujita H, Tanigaki T, Shimonaga T, Ueoka A, Tsubakimoto Y, Sakatani T, Kimura S, Inoue K and Kitamura M. Clinical implications of coronary pressure measurement after stent implantation. *Cardiovasc Interv Ther*. 2013;28:170-7.
5. Ito T, Tani T, Fujita H and Ohte N. Relationship between fractional flow reserve and residual plaque volume and clinical outcomes after optimal drug-eluting stent implantation: insight from intravascular ultrasound volumetric analysis. *Int J Cardiol*. 2014;176:399-404.
6. Johnson NP, Toth GG, Lai D, Zhu H, Acar G, Agostoni P, Appelman Y, Arslan F, Barbato E, Chen SL, Di Serafino L, Dominguez-Franco AJ, Dupouy P, Esen AM, Esen OB, Hamilos M, Iwasaki K, Jensen LO, Jimenez-Navarro MF, Katritsis DG, Kocaman SA, Koo BK, Lopez-Palop R, Lorin JD, Miller LH, Muller O, Nam CW, Oud N, Puymirat E, Rieber J, Rioufol G, Rodes-Cabau J, Sedlis SP, Takeishi Y, Tonino PA, Van Belle E, Verna E, Werner GS, Fearon WF, Pijls NH, De Bruyne B and Gould KL. Prognostic value of fractional flow reserve: linking physiologic severity to clinical outcomes. *J Am Coll Cardiol*. 2014;64:1641-54.
7. Doh JH, Nam CW, Koo BK, Lee SY, Choi H, Namgung J, Kwon SU, Kwak JJ, Kim HY, Choi WH and Lee WR. Clinical Relevance of Poststent Fractional Flow Reserve After Drug-Eluting Stent Implantation. *J Invasive Cardiol*. 2015;27:346-51.
8. Reith S, Battermann S, Hellmich M, Marx N and Burgmaier M. Correlation between OCT-derived intrastent dimensions and fractional flow reserve measurements after coronary stent implantation and impact on clinical outcome. *J Invasive Cardiol*. 2015;27:222-8.
9. Agarwal SK, Kasula S, Hacioglu Y, Ahmed Z, Uretsky BF and Hakeem A. Utilizing Post-Intervention Fractional Flow Reserve to Optimize Acute Results and the Relationship to Long-Term Outcomes. *JACC Cardiovasc Interv*. 2016;9:1022-31.
10. Kasula S, Agarwal SK, Hacioglu Y, Pothineni NK, Bhatti S, Ahmed Z, Uretsky B and Hakeem A. Clinical and prognostic value of poststenting fractional flow reserve in acute coronary syndromes. *Heart (British Cardiac Society)*. 2016;102:1988-1994.
11. Matsuda J, Murai T, Kanaji Y, Usui E, Araki M, Niida T, Ichijyo S, Hamaya R, Lee T, Yonetsu T, Isobe M and Kakuta T. Prevalence and Clinical Significance of Discordant Changes in Fractional and Coronary Flow Reserve After Elective Percutaneous Coronary Intervention. *J Am Heart Assoc*. 2016;5:e00400.
12. Li SJ, Ge Z, Kan J, Zhang JJ, Ye F, Kwan TW, Santoso T, Yang S, Sheiban I, Qian XS, Tian NL, Rab TS, Tao L and Chen SL. Cutoff Value and Long-Term Prediction of Clinical Events by FFR Measured Immediately After Implantation of a Drug-Eluting Stent in Patients With Coronary Artery Disease: 1- to 3-Year Results From the DKCRUSH VII Registry Study. *JACC Cardiovasc Interv*. 2017;10:986-995.
13. Piroth Z, Toth GG, Tonino PAL, Barbato E, Aghlmandi S, Curzen N, Rioufol G, Pijls NHJ, Fearon WF, Juni P and De Bruyne B. Prognostic Value of Fractional Flow Reserve Measured Immediately After Drug-Eluting Stent Implantation. *Circ Cardiovasc Interv.* 2017;10: e005233.
14. Lee JM, Hwang D, Choi KH, Rhee TM, Park J, Kim HY, Jung HW, Hwang JW, Lee HJ, Jang HJ, Kim SH, Song YB, Cho YK, Nam CW, Hahn JY, Shin ES, Kawase Y, Matsuo A, Tanaka N, Doh JH, Koo BK and Matsuo H. Prognostic Implications of Relative Increase and Final Fractional Flow Reserve in Patients With Stent Implantation. *JACC Cardiovasc Interv*. 2018;11:2099-2109.
15. Usui E, Murai T, Kanaji Y, Hoshino M, Yamaguchi M, Hada M, Hamaya R, Kanno Y, Lee T, Yonetsu T and Kakuta T. Clinical significance of concordance or discordance between fractional flow reserve and coronary flow reserve for coronary physiological indices, microvascular resistance, and prognosis after elective percutaneous coronary intervention. *EuroIntervention*. 2018;14:798-805.
16. Azzalini L, Poletti E, Demir OM, Ancona MB, Mangieri A, Giannini F, Carlino M, Chieffo A, Montorfano M, Colombo A and Latib A. Impact of Post-Percutaneous Coronary Intervention Fractional Flow Reserve Measurement on Procedural Management and Clinical Outcomes: The REPEAT-FFR Study. *J Invasive Cardiol*. 2019;31:229-234.
17. Hakeem A, Ghosh B, Shah K, Agarwal S, Kasula S, Hacioglu Y, Bhatti S, Ahmed Z and Uretsky B.

Incremental Prognostic Value of Post-Intervention Pd/Pa in Patients Undergoing Ischemia-Driven Percutaneous Coronary Intervention. *JACC Cardiovasc Interv*. 2019;12:2002-2014.
18. Hoshino M, Kanaji Y, Hamaya R, Kanno Y, Hada M, Yamaguchi M, Sumino Y, Usui E, Sugano A, Murai T, Lee T, Yonetsu T and Kakuta T. Prognostic value of post-intervention fractional flow reserve after intravascular ultrasound-guided second-generation drug-eluting coronary stenting. *EuroIntervention*. 2019;15:e779-e787.
19. Hwang D, Lee JM, Lee HJ, Kim SH, Nam CW, Hahn JY, Shin ES, Matsuo A, Tanaka N, Matsuo H, Lee SY, Doh JH and Koo BK. Influence of Target Vessel on Prognostic Relevance of Fractional Flow Reserve After Coronary Stenting. *EuroIntervention*. 2019;15(5):457-464.
20. Yang HM, Lim HS, Yoon MH, Seo KW, Choi BJ, Choi SY, Hwang GS and Tahk SJ. Usefulness of the trans-stent fractional flow reserve gradient for predicting clinical outcomes. *Catheter Cardiovasc Interv*. 2020;95:E123-e129.
21. Hwang D, Lee JM, Yang S, Chang M, Zhang J, Choi KH, Kim CH, Nam CW, Shin ES, Kwak JJ, Doh JH, Hoshino M, Hamaya R, Kanaji Y, Murai T, Zhang JJ, Ye F, Li X, Ge Z, Chen SL, Kakuta T and Koo BK. Role of Post-Stent Physiological Assessment in a Risk Prediction Model After Coronary Stent Implantation. *JACC Cardiovasc Interv*. 2020;13:1639-1650.
22. Hamaya R, Mittleman MA, Hoshino M, Kanaji Y, Murai T, Lee JM, Choi KH, Zhang J, Ye F, Li X, Chen S and Kakuta T. Prognostic Value of Pre-Revascularization Fractional Flow Reserve Mediated by the Post-Revascularization Level: A Causal Mediation Analysis. *JAMA Network Open*. 2020;3(9):e2018162.
23. Shin D, Lee SH, Lee JM, Choi KH, Hwang D, Lee HJ, Jang HJ, Kim HK, Kwak JJ, Ha SJ, Song YB, Shin ES and Doh JH. Prognostic Implications of Post-Intervention Resting Pd/Pa and Fractional Flow Reserve in Patients With Stent Implantation. *JACC Cardiovasc Interv*. 2020;13:1920-1933.
24. Hokama Y, Tanaka N, Takashima H, Kadota K, Fujita H, Tan M, Yamada R, Naruse H, Kawamura A, Suzuki N, Takeuchi T, Tazaki J, Yamaki M, Takamisawa I, Abe S, Terai H, Makiguchi N, Matsumoto C and Chikamori T. Insufficient recovery of fractional flow reserve even after optimal implantation of drug-eluting stents: 3-year outcomes from the FUJI study. *J Cardiol*. 2020;S0914-5087(20)30397-X.
25. Shin D, Dai N, Lee SH, Choi KH, Lefieux A, Molony D, Hwang D, Kim HK, Jeon KH, Lee HJ, Jang HJ, Ha SJ, Park TK, Yang JH, Song YB, Hahn JY, Choi SH, Doh JH, Shin ES, Nam CW, Koo BK, Gwon HC, Ge J and Lee JM. Physiological Distribution and Local Severity of Coronary Artery Disease and Outcomes After Percutaneous Coronary Intervention. *JACC Cardiovasc Interv*. 2021;14:1771-1785.
26. Yang S, Zhang J, Hwang D, Lee JM, Nam CW, Shin ES, Doh JH, Hoshino M, Hamaya R, Kanaji Y, Murai T, Zhang JJ, Ye F, Li X, Ge Z, Chen SL, Kakuta T and Koo BK. Effect of Coronary Disease Characteristics on Prognostic Relevance of Residual Ischemia After Stent Implantation. *Front Cardiovasc Med*. 2021;8:696756.
27. Zhang J, Hwang D, Yang S, Kim CH, Lee JM, Nam CW, Shin ES, Doh JH, Hoshino M, Hamaya R, Kanaji Y, Murai T, Zhang JJ, Ye F, Li X, Ge Z, Chen SL, Kakuta T and Koo BK. Differential Prognostic Implications of Pre- and Post-Stent Fractional Flow Reserve in Patients Undergoing Percutaneous Coronary Intervention. *Korean Circ J*. 2022;52:47-59.
28. Diletti R, Masdjedi K, Daemen J, van Zandvoort LJC, Neleman T, Wilschut J, Den Dekker WK, van Bommel RJ, Lemmert M, Kardys I, Cummins P, de Jaegere P, Zijlstra F and Van Mieghem NM. Impact of Poststenting Fractional Flow Reserve on Long-Term Clinical Outcomes: The FFR-SEARCH Study. *Circ Cardiovasc Interv.* 2021;14:e009681.
29. Collison D, Didagelos M, Aetesam-Ur-Rahman M, Copt S, McDade R, McCartney P, Ford TJ, McClure J, Lindsay M, Shaukat A, Rocchiccioli P, Brogan R, Watkins S, McEntegart M, Good R, Robertson K, O'Boyle P, Davie A, Khan A, Hood S, Eteiba H, Berry C and Oldroyd KG. Post-stenting fractional flow reserve vs coronary angiography for optimization of percutaneous coronary intervention (TARGET-FFR). *Eur Heart J*. 2021;42:4656-4668.

© 2022 Hwang D et al. *JAMA Network Open.*
